# Addressing the different paces of climate and air quality combustion emissions across the world

**DOI:** 10.1016/j.isci.2023.108686

**Published:** 2023-12-07

**Authors:** Fabio Monforti-Ferrario, Monica Crippa, Enrico Pisoni

**Affiliations:** 1 European Commission, Joint Research Centre (JRC), Ispra, VA, Italy

**Keywords:** Environmental science, Environmental policy, Energy policy

## Abstract

Greenhouse gases (GHG) and air pollutants (AP) share several anthropic sources but evolve differently in time across the various regions of the globe. Fossil and biological fuel combustion is by far the single process producing the highest amounts of both types of compounds. We have analyzed the paces of change of both GHG and AP emissions across the world and in some selected highly emitting regions using purposely designed indicators.

We have observed that, overall, combustion processes are generally producing a lower amount of pollutants per unit of GHG emitted in 2018 than in 1970, with the noticeable exception of ammonia emissions in transport. Nevertheless, comparing countries at different development levels, evidence of possible further improvement clearly emerges, depending on the technological evolution of the most important emitting sectors and on the implementation of appropriate control measures and policies.

## Highlights

• We compared the combustion related greenhouse gases and air pollutants emissions
• Air pollutants per unit of greenhouse gases show a generally decreasing trend
• Exceptions exist, which will deserve further investigation
• Comparing across stages of development, room for improvement clearly emerges

## Graphical abstract

**Figure:**
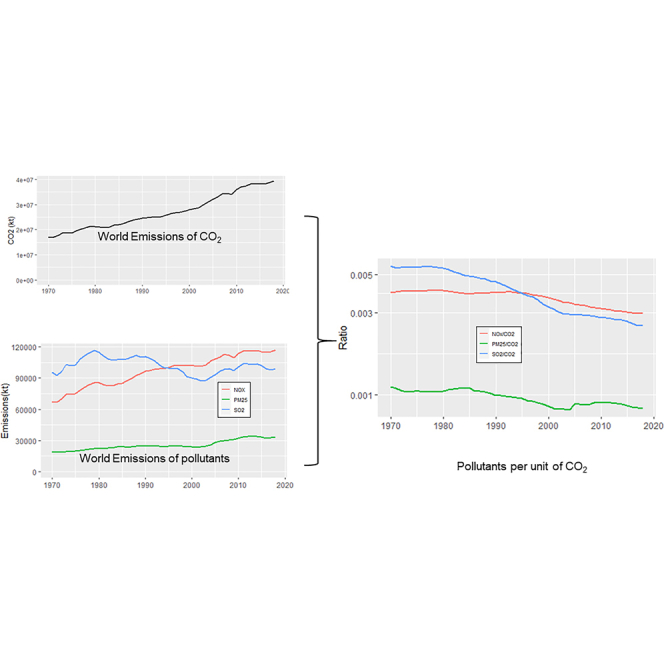

## Introduction

A large number of human activities cause the emissions of various types of gases and aerosols in the atmosphere, including both greenhouse gases (GHG) and air pollutants (AP). Scientific literature has investigated the factors impacting on the actual value of emissions at the national level: according to the classical Kaya identity approach^1^ population, GDP, and energy consumption play a major role in determining the GHG emissions of a country. On top of these major drivers, other aspects also influence the overall emissions. For instance, the energy mix and the technological mix of the country,^2^ the effective deployment of appropriate end-of-pipe technologies, the proper implementation of emissions control legislation and, more generally, the policy framework put in place by a country to tackle GHG and AP emissions.^3^

The cited factors do not necessarily impact GHG and AP emissions in the same way, as changes in GHG emissions are not in principle mirrored by comparable changes in AP emissions and vice versa: more precisely, some changes in emission drivers impact both GHG and AP emissions to a similar extent, while others do not. Synergies happen when control policies aimed at decreasing GHG emissions lead to AP decreasing too and vice versa. As an example of a synergetic effect, the energy saving measures taken at the urban level have been demonstrated to produce co-benefits, leading to a decrease in both the AP and GHG emissions.^4^

On the contrary, trade-offs happen when an action aimed at reducing GHG emissions results in an increase in emissions of some pollutants: as an example, the use of biomass based solid and liquid fuels for power production is generally beneficial in terms of GHG emissions, but could negatively impact the air quality because of its relatively high AP emission factors, especially if associated with less advanced technologies, notably in the domestic sector^5^^,^^6^*.* From a global policy perspective, it has been shown that climate mitigation and air quality policies provide overall mutual benefits each other on the global scale^7^ and positive synergies have played a more relevant role than negative trade-offs. Nevertheless, even when behaving synergically, the time evolution and the actual pace of change of AP and GHG emissions can be very different.

In this article, we investigate how AP and GHG emissions have changed their relative magnitudes starting from 1970 to 2018 across the world’s nations and regions and suggest some possible interpretations of their pathways in terms of technological changes and policy implementation.

To answer this research question, we have focused our attention on the amount of air pollutants emitted per unit of GHG emitted in specific sectors and regions. In our opinion, such an indicator allows a direct comparison of the relative evolution of air pollution and GHG emissions and, from a policy design perspective, it is a very useful tool to distinguish sectors and regions where positive synergies between climate and air quality policies are taking place from sectors and regions where the two types of emissions are more clearly decoupled. To our knowledge, this study is the first to propose the use of such an indicator and to compute and analyze it for the most relevant pollutants.

We decided to concentrate our analysis on combustion, i.e., the physical process currently leading to the largest large share of both key AP emissions and CO_2_ – currently the most impacting GHG.^8^ Overall, according to the EDGAR (Emissions Database for Global Atmospheric Research)^9^^,^^10^^,^^11^ fuel combustion processes, coded as 1.A in the IPCC nomenclature,^12^ in 2018 have originated 88.2% of CO_2_ emissions, 94.5% of NO_x_ emissions, 80.7% of PM_2.5_ emissions and 95.6% of SO_2_ at the global scale.

In order to compare the evolution of GHG and APs emissions sector by sector, we have built sets of indicators for the whole combustion macro sector and for its most relevant sectors, as detailed in Table 1, with the overall objective of investigating the human activities at the origin of the largest shares of both GHGs and air pollutants.

**Table 1:** Columns 1 and 2: sectors and sub-sectors considered in this study

Macro Sector	IPCC code	Indicators	Shares of world total emissions in 2018
Fuel combustion	1.A	PM_2.5_/CO_2_	CO_2_ – 88.2%
		NO_x_/CO_2_	PM_2.5_–80.7%
		SO_2_/CO_2_	NO_x_ – 94.5%
			SO_2_ – 95.6%

**Sector**	**IPCC code**	**Indicators**	**Shares of world total emissions in 2018**

Energy Industry	1.A.1	PM_2.5_/CO_2_	CO_2_ – 36.2%
		NO_x_/CO_2_	PM_2.5_–18.2%
		SO_2_/CO_2_	NO_x_ – 29.9%
			SO_2_ – 46.2%
Manufacturing and construction	1.A.2	PM_2.5_/CO_2_	CO_2_ – 18.5%
		NO_x_/CO_2_	PM_2.5_–22.5%
		SO_2_/CO_2_	NO_x_ – 17.6%
			SO_2_ – 29.9%
Transport	1.A.3	PM_2.5_/CO_2_	CO_2_ – 18.8%
		NO_x_/CO_2_	PM_2.5_–9.3%
		SO_2_/CO_2_	NO_x_ – 42.4%
		NH_3_/CO_2_	SO_2_ – 12.9%
			NH_3_ – 1.5%
Buildings	1.A.4	PM_2.5_/CO_2_	CO_2_ – 14.2%
		NO_x_/CO_2_	PM_2.5_–29.8%
		SO_2_/CO_2_	NO_x_ – 4.3%
			SO_2_ – 5.9%

Column 3: indicators selected for each sector/sub-sector. Column 4: shares of world emissions attributable to each sector/subsector for the pollutants and GHG used to build the indicators.

As shown in Table 1, for each sector, we have analyzed the trends of three indicators, namely PM_2.5_/CO_2_, NO_x_/CO_2_, and SO_2_/CO_2_. Although sector 1.A.3 (transport) is responsible for a very limited share of NH_3_ emissions, we have also included for this sector a fourth indicator, NH_3_/CO_2_, in order to take into consideration the emerging issue of ammonia emissions in transport. Indeed, in vehicles equipped with a three-way catalyst NH_3_ is a side product in the NO_x_ reduction process and has been shown to be an important and recently increasing source of NH_3_ in urban environments.^13^

It is worth precising that in our analysis we have included the emissions of CO_2_ and APs originating from both fossil fuels and fuels of biological origin (e.g., wood, biogas, biofuels, and so forth) because of the well known impact of biomass burning on the global air pollution.

The time series of indicators listed in Table 1 have been computed based on the latest releases of EDGAR for both GHG^9^ and air pollutants,^10^ from 1970 to 2018, the latest year included in both datasets. Given its long-term perspective and thanks to its consistent methodology, EDGAR is a suitable tool for investigating decadal trends of both GHG and air pollutant emissions, also allowing meaningful comparisons across substances, sectors, and geographical areas. In retrospective, the past evolution of these indicators could support the comparison of the relative effectiveness of actions aimed at either climate change mitigation or air quality protection across different economic sectors and geographical regions.

It is also worth observing that the indicators used in this study are also equivalent to the ratios between sector-specific Implied Emission Factors (IEF) for air pollutants and IEF for GHG, as shown in more detail in the methodology section.

Having this in mind, a simple but effective interpretation of our results follows: a decreasing indicators trend identifies periods and regions where policy choices, technological evolution, fuel mix evolution, and the appropriate deployment of end of pipe abatement technologies have led to an effective decoupling of air pollution from GHG growth (or a slight decrease in some cases). In this situation, the combustion process has become “cleaner” from an air pollution perspective, even when and where both types of emissions have overall increased.

On the contrary, an increasing indicators trend could identify periods and regions where there is a risk that more attention is given to CO_2_ control than to continuously addressing air quality issues and combustion processes have become “dirtier” in terms of pollutants emitted per unit of GHG.

In practice, we have computed, using EDGAR data, the full time series for all indicators listed in Table 1 in different regions of the world. We have focused our attention on the European Union and China, following our previous investigations on the impacts of technological changes and regulatory frameworks on air pollution,^2^ together with a more general perspective, on the larger groups of Former Annex I (FAI) and Former Non-Annex I (FNAI) countries, similarly to another of our previous analysis.^3^ Finally, also world average values of the indicators have also been computed.

Besides the already cited novelty in the proposed indicators, we also would like to emphasize that, to our knowledge, this is the frirst study where AP emissions per unit of GHG are compared among several different areas of the world and along such an extended time span.

## Results

Tables 2, 3, 4, and 5 show the absolute values of the selected indicators in 2018, for the sectors and regions considered. Tables 2, 3, 4, and 5 allow a direct comparison of the current quality of combustion across sectors and regions in terms of amount of pollutants per unit of CO_2_ emitted and will be discussed in more detail in the next paragraph.

**Table 2:** Values of SO_2_/CO_2_ ratio in 2018 for selected IPCC sectors and world regions

SO_2_/CO_2_	EU27	China	FAI	FNAI	World
**1.A**	1.11	2.80	1.36	2.88	2.52
**1.A.1**	1.63	1.52	2.13	3.43	2.96
**1.A.2**	1.65	5.69	1.78	4.39	3.74
**1.A.3**	0.26	1.52	0.28	0.61	1.60
**1.A.4**	0.73	2.15	0.67	1.13	0.97

Units: kgSO_2_/tCO_2_.

**Table 3:** Values of PM_2.5_/CO_2_ ratio in 2018 for selected IPCC sectors and world regions

PM_2.5_/CO_2_	EU27	China	FAI	FNAI	World
**1.A**	0.32	1.21	0.25	1.14	0.83
**1.A.1**	0.07	0.87	0.07	0.67	0.46
**1.A.2**	0.52	1.57	0.50	1.30	1.11
**1.A.3**	0.18	0.61	0.19	0.41	0.45
**1.A.4**	0.32	1.21	0.58	2.57	1.91

Units: kgPM_2.5_/tCO_2_.

**Table 4:** Values of NO_x_/CO_2_ ratio in 2018 for selected sectors and world regions

NO_x_/CO_2_	EU27	China	FAI	FNAI	World
**1.A**	1.75	2.71	2.06	2.86	2.97
**1.A.1**	1.07	2.28	1.59	2.68	2.29
**1.A.2**	1.97	2.96	2.09	2.82	2.64
**1.A.3**	3.39	6.33	3.34	6.21	6.26
**1.A.4**	0.91	1.00	1.02	0.75	0.84

Units: kgNO_x_/tCO_2_.

**Table 5:** Values of NH_3_/CO_2_ ratio in 2018 in the transport sector (IPCC 1.A.3) and selected world regions

NH_3_/CO_2_	EU27	China	FAI	FNAI	World
**1.A.3**	0.167	0.099	0.148	0.087	0.104

Units: kgNH_3_/tCO_2_.

As previously anticipated, thanks to the long time series provided by EDGAR we have computed the full set of trends for the selected indicators from 1970 to 2018.

**Figure 1:**
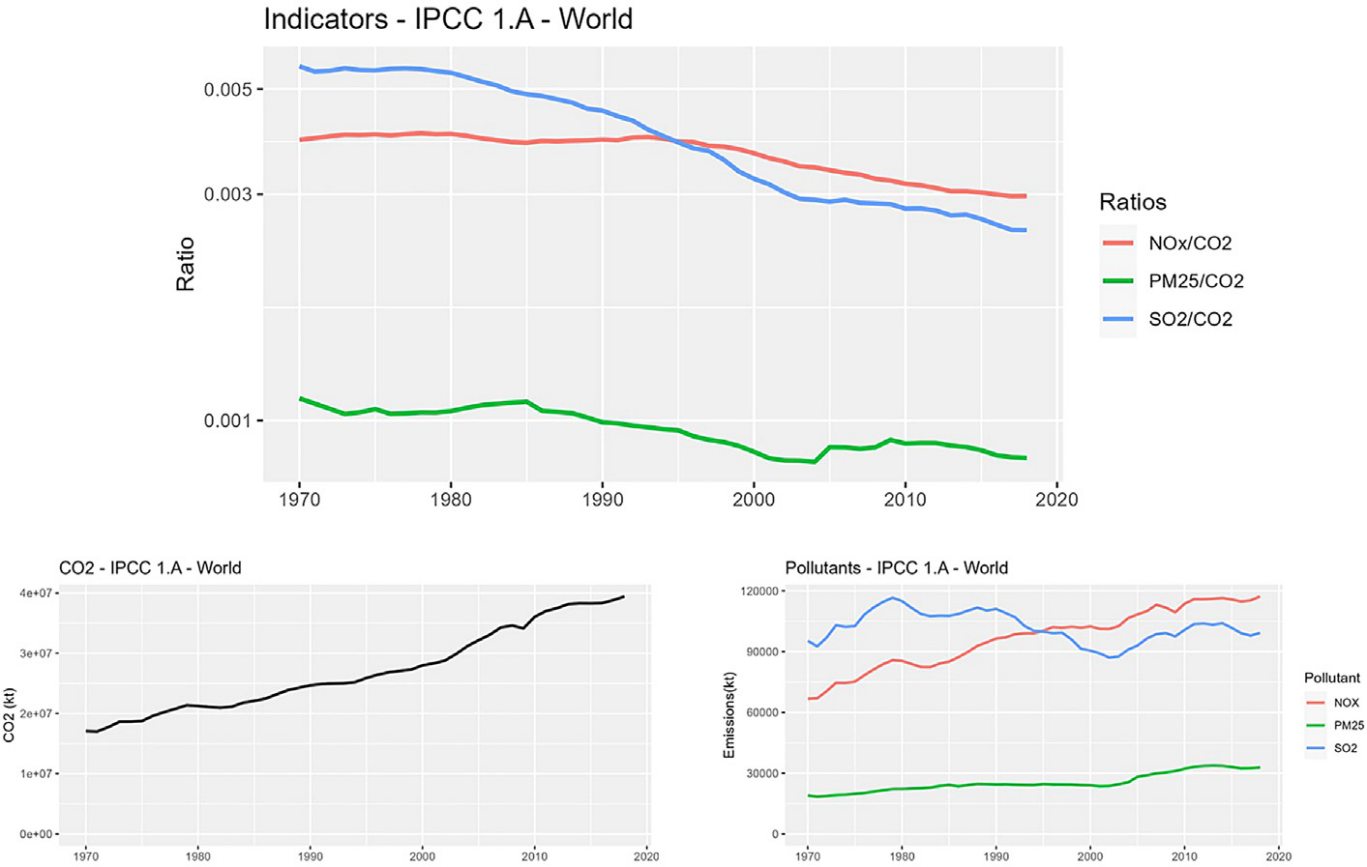
Top panel: Time series (1970–2018) of selected indicators in the IPCC 1.A (Combustion) sector in all world Bottom left: CO_2_ emissions (1970–2018) in 1.A sector in in all world. Bottom right: emissions (1970–2018) of selected pollutants in 1.A sector in in all world.

Figure 1 shows these trends for the world and the whole 1.A sector together with the time series of both AP and CO_2_ world emissions of the same sector. More in detail, the figure is composed of an upper panel with the time series of the selected indicators, while the bottom left and the bottom right graphs show the time series of world emissions of CO_2_ and of pollutants respectively for sector 1.A.

The full set of trends is shown in Figures S1–S24 of the supplementary material, together with the absolute values of the concerned air pollutants and of CO_2_ emissions from 1970 to 2018 in the analyzed sectors and regions in a similar format. Key features emerging from Figures 1 and S1–S24 will also be discussed in next paragraph.

Tables 6, 7, 8, and 9 provide a more concise view of the indicator trends, allowing us to identify some key aspects to be also deepened in next paragraph. For each of the selected indicators, the tables show its percentage change between its decadal average computed for the time period (2009–2018) and the two reference decadal average considering (1970–1979) and (1990–1999) respectively, for the IPCC sectors and regions investigated.

**Table 6:** Percentage changes of SO_2_/CO_2_ ratio between its latest available decadal average (2009–2018) and the reference averages (1970–1979) and (1990–1999) for selected sectors and regions

SO_2_/CO_2_	EU27		China		FAI		NFAI		World	
Reference	1970–79	1990–99	1970–79	1990–99	1970–79	1990–99	1970–79	1990–99	1970–79	1990–99
**1.A**	−81%	−65%	−41%	−45%	−74%	−54%	−29%	−30%	−51%	−32%
**1.A.1**	−81%	−71%	−80%	−78%	−75%	−56%	−65%	−54%	−66%	−49%
**1.A.2**	−69%	−41%	−34%	−29%	−64%	−36%	−16%	−14%	−24%	−7%
**1.A.3**	−84%	−47%	−65%	−52%	−80%	−47%	−73%	−41%	−45%	−11%
**1.A.4**	−86%	−58%	−23%	−13%	−84%	−59%	−37%	−30%	−69%	−40%

**Table 7:** Percentage changes of PM_2.5_/CO_2_ ratio between its latest available decadal average (2009–2018) and the reference averages (1970–1979) and (1990–1999) for selected sectors and regions

PM_2.5_/CO_2_	EU27		China		FAI		NFAI		World	
Reference	1970–79	1990–99	1970–79	1990–99	1970–79	1990–99	1970–79	1990–99	1970–79	1990–99
**1.A**	−44%	−23%	−51%	−40%	−55%	−22%	−41%	−28%	−17%	−8%
**1.A.1**	−79%	−60%	−54%	−34%	−80%	−43%	−32%	−24%	−3%	17%
**1.A.2**	−12%	−7%	−18%	−21%	−16%	3%	−2%	−4%	31%	14%
**1.A.3**	−49%	−48%	33%	−23%	−49%	−34%	−30%	−35%	−26%	−16%
**1.A.4**	−20%	5%	−29%	−23%	−30%	−7%	−19%	−10%	−5%	−5%

**Table 8:** Percentage changes of NO_x_/CO_2_ ratio between its latest available decadal average (2009–2018) and the reference averages (1970–1979) and (1990–1999) for selected sectors and regions

NO_x_/CO_2_	EU27		China		FAI		FNAI		World	
Reference	1970–79	1990–99	1970–79	1990–99	1970–79	1990–99	1970–79	1990–99	1970–79	1990–99
**1.A**	−44%	−42%	26%	−3%	−44%	−42%	5%	−7%	−23%	−21%
**1.A.1**	−59%	−50%	−45%	−41%	−39%	−31%	−28%	−24%	−18%	−16%
**1.A.2**	−22%	−12%	4%	−10%	−17%	−5%	4%	1%	3%	4%
**1.A.3**	−67%	−54%	−50%	−40%	−68%	−61%	−45%	−34%	−48%	−38%
**1.A.4**	−12%	−5%	40%	28%	−6%	−2%	17%	9%	−3%	2%

**Table 9:** Percentage changes of NH_3_/CO_2_ ratio between its latest available decadal average (2009–2018) and the reference averages (1970–1979) and (1990–1999) in the Transport sector (IPCC 1.A.3) and selected regions

NH_3_/CO_2_	EU27		China		FAI		FNAI		World	
Reference	1970–79	1990–99	1970–79	1990–99	1970–79	1990–99	1970–79	1990–99	1970–79	1990–99
**1.A.3**	1675%	64%	2520%	524%	1066%	171%	403%	136%	647%	131%

In Tables 6, 7, 8, and 9 positive values identify areas and sectors where combustion processes have become “dirtier” in the last available decade (2009–2018) in terms of pollutant emission per unit of emitted CO_2_ in comparison with the reference decades (either 1970–79 or 1990–99). On the contrary, negative values refer to sectors and regions where the combustion processes have become “cleaner” again in terms of pollutants per unit of CO_2_ emitted.

Tables 6, 7, 8, and 9 practically allow a simplified comparison of sometimes quite complex trends, fully shown in Figures 1 and S1–S24, to which the reader is referred for more details.

Finally, Tables S1–S4 report the Pearson’s correlation coefficients between GHG and AP time series in the selected regions and sectors, providing further insights for trend analysis. More in detail, given that the variation range of correlation coefficients is by definition limited between −1 and 1, high positive correlation values indicate a synchronous behavior between GHG and AP emissions. On the contrary, lower and negative correlation values indicate progressively higher decoupling^14^^,^^15^^,^^16^ between the AP and GHG trends.

### Results interpretation

Tables 2, 3, 4, 5, 6, 7, 8, and 9, Figures 1, S1–S24 and Tables S1–S4 show some features that in our opinion are worth to be noticed and reported.

• Absolute values and differences among regions (Tables 2, 3, 4, and 5)

All the indicators involving PM_2.5_, NO_x_, and SO_2_ are generally higher in China and FNAI countries in comparison with EU27 and FAI countries, in 2018 (Tables 2, 3, and 4). Although in some cases the differences across regions seem to decrease with time, other cases contradict such a view, and a large difference remains evident. It is worth reminding once again that several factors concur to create this difference: fuel mix, technological mix, deployment and effectiveness of end of pipe technologies and also the possible different weight of sub-sub-sectors. For instance the sub-sector 1.A.1 is the union of three sub-sub-sectors (namely 1.A.1.A – Electricity and Heat Generation, 1.A.1.B – Petroleum Refining and 1.A.1.C – Manufacture of Solid Fuels) possibly having different relative importance in different areas. Nevertheless, our previous studies^2^^,^^3^ have shown the importance of both technological and regulatory landscapes for controlling air pollution emissions.

For this reason, it seems reasonable to suppose that such an important difference in the indicators studied could suggest that in FNAI countries an important decrease in pollution emissions could be pursued by further aligning the technology mix and regulations with FAI countries.

A major exception to this general picture is the value of SO_2_/CO_2_ in the 1.A.1 sector (power production) in China, which has recently become close to the correspondent EU indicator, confirming that convergence between FAI and FNAI countries is indeed possible and indicating a good performance in the sector desulfurisation in China.

The NH_3_/CO_2_ indicator for the transport sector (1.A.3) is on the contrary higher in EU27 and FAI than in China and FNAI: NH_3_ per unit of CO_2_ emitted in 2018 is 41% lower in FNAI than in FAI. The reason for this different behavior is likely to be found in the fact that NH_3_ is emitted as a side product of NO_x_ reduction in the three-ways catalytic systems,^13^^,^^17^ still less common in FNAI than in FAI, where in most cases they have become compulsory for new vehicles entering the market since decades (e.g., since 1975 in the US and since the 1993 Euro-1 regulation in the EU).

• Temporal trends (Tables 6, 7, 8, and 9; Figure 1; S1–S24, and Tables S1–S4)

In EU27 and more generally in FAI countries, the indicators involving PM_2.5_, NO_x,_ and SO_2_ have improved in the long term since 1970 (Tables 6, 7, and 8), sometimes by very relevant percentages. Figures such as S.1 or S.3 show in more detail how the ratios between AP and GHG emissions have been generally decreasing from 1970 on, reaching a plateau in the period 2005–2010 and remaining broadly constant thereafter. The overall trend depends on the pollutant considered, with SO_2_/CO_2_ generally being the indicator showing the most relevant decrease.

In EU27 and FAI countries, SO_2_/CO_2_ and NO_x_/CO_2_ indicators generally show a decreasing trend, also in the shorter term, between 2005 and 2018. On the contrary, PM_2.5_/CO_2_ shows early signs of a slower decreasing or even an upward trend in many 1.A sub-sectors (see Table 7) indicating a convex shape of the trend (See for instance Figures S5, S10, and S20), reaching a minimum around the years 2000–2005 and then slightly increasing.

For the overall combustion processes, both in China and FNAI countries (Figures S2 and S4), most indicators show an early increasing phase (or a plateau in the case of PM_2.5_/CO_2_), followed, after about 1990, by a decrease still taking place in 2018. No signs of plateaus or tendency inversion are visible, differently from what has been observed in EU27 and FAI.

Again, in China and FNAI countries, SO_2_/CO_2_ shows also important decreases both in the long and short term, with the exception of a slight rebound in the short term in China for sector 1.A.4 (residential) – See Figures S21.

In the same areas, PM_2.5_/CO_2_ is still on a clearly decreasing pattern (see e.g., Figures S2 and S4), and the convex shape seen in EU and FAI countries has not manifested here. Nevertheless, the absolute value of this indicator in China and FNAI is still about three times higher than in FAI and EU27 on average, indicating the important room for improvement, as already previously discussed.

Remaining in China and FNAI countries, NO_x_/CO_2_ shows an upward trend in sub sector 1.A.4 (Table 8; Figures S21 and S23), although also overall still decreasing both for the whole combustion sector (Figures S2 and S4) and in most of its sub-sectors (see e.g., Figures S6, S8, S16 and S18), suggesting a special care should be put into keeping the combustion in residential sectors efficient in these regions.

The NH_3_/CO_2_ indicator for the transport sector (1.A.3 – Table 9) has increased by very important amounts in all regions although its most recent trend shows a slow down of the increase or even some decreases.

It is finally worth mentioning that, quite interestingly, similar trends of the indicators sometimes originate from opposite underlying emission patterns.

For instance, SO_2_/CO_2_ has decreased in the EU27 and in China in all sectors investigated (see Table 6). Nevertheless, in the EU27 both SO_2_ and CO_2_ emissions have shown overall decreasing or sometimes constant trends in latest decades (see e.g., the bottom panels of Figures S1, S5, S10, and S20); on the contrary, in China, both SO_2_ and CO_2_ emissions have generally increased at least until 2000 (see e.g., the bottom panels of Figures S2, S6 and S11).

A closest observation of Tables S1–S4 confirms the different underlying nature of apparently similar indicator trends. Indeed Pearson’s correlation coefficients for all pollutants except NH_3_ are generally higher in China and FNAI countries than in the EU and FAI, evidencing a later or less pronounced decoupling of AP and GHG in these regions. Negative values for correlation coefficients also appear in EU and FAI only, with especially relevant negative values reported for the transport sector (1.A.3) for SO_2_ and NO_x_.

On the contrary, higher values for Pearson’s correlation coefficients for NH_3_ in all regions confirms a still strong coupling with CO_2_ emissions everywhere in the world.

## Discussion

The observed trends and values of the indicators shown suggest some interpretations.

• In all sectors, long standing policies against SO_2_ emissions, for instance, the implementation of control measures under the UNECE Convention on Long-range Transboundary Air Pollution^18^^,^^19^ have been extremely effective. Even in context of increasing AP and CO_2_ emissions an overall decrease of SO_2_/CO_2_ is evident in all regions studied. On average in the whole world, combustion processes (1.A) emit in 2018 less than half of the SO_2_ per unit of CO_2_ emitted in 1970. The most relevant progress took place in EU27 where in 2018, the SO_2_ emissions per unit of CO_2_ in the European power sector (1.A.1) amounted to 17% of the 1970 value and to 13% of the 1970 value in the transport sector.
In China SO_2_ emissions, starting to show a decrease after early 2010s following the introduction stringent emission limits on road vehicles and power plants^2^ have decoupled from CO_2_ growth since 1990s (see Figures S2) with current values of SO_2_ emissions per unit of CO_2_ in the overall 1.A sector being 57% lower than its peak value.
This analysis confirms how fossil fuel combustion has become more and more “sulfur free” all across the world: both the adoption of low-sulphur fuels (e.g., natural gas and solid biomass) in energy and desulphurized oil in transport and the application of effective end-of-pipe technologies are at the basis of such a result.
• Similarly, although to a more limited extent, both NO_x_/CO_2_ and PM_2.5_/CO_2_ show the effect of technologies and control policies focusing on the pollutants emissions that seem to have efficiently decoupled GHG and air pollutants trends in most of regions and sectors.
• Nevertheless, in some sub-sectors the overall decrease of pollutants versus CO_2_ emissions ratio seems to have reached a plateau. This could be due to both an increased emphasis in recent years on GHG policies in comparison with air pollution control and by the fact that air pollution control technologies have reached, at least in some regions, very high efficiency standards and penetration rates, difficult to be further overcome. The early signs of a tendency toward inversion, with indicators seemingly increasing in several FAI countries fortify both possible explanations.
• These early signs of rebound in some indicators that we have observed in some areas (e.g., for PM_2.5_/CO_2_ in the EU27 and in the FAI countries) deserve attention: it is important that in the rush for reaching more and more stringent GHG abatement targets, air pollution control remains also a key objective. Actions contrasting GHG and air pollution have generally proceeded hand in hand, with reductions in air pollution even anticipating upcoming GHG controls. A reverse of such a situation is evidently not desirable, especially when ambitious targets for both GHG and air pollution have to be pursued, as it is the case for the zero pollution and the zero emissions targets set by the EU.^20^^,^^21^ Mitigation and air pollution control actions should continue to be seen as synergetic in order to exploit co-benefits to the largest possible extent. For instance, the use of biomass-based fuels should take place with very high attention to related air quality emissions, to avoid negative spill-overs of mitigation policies to air quality levels.^22^
• The sharply increasing trend of NH_3_/CO_2_ from transport in all regions deserves also a very special attention: cathalysis technologies are among the known causes of such an increase^13^: although transport remains a minor source of ammonia, its emissions take place in urban centers where they contribute to the formation of secondary aerosols.^23^

In conclusion, we have analyzed the relative changes of AP and GHG combustion-caused emissions in the period 1970–2018 for the whole world and some regions and countries (namely FNAI, FAI, EU27 and China). Both AP and GHG changes clearly depend on the technological evolution of the most important emitting sectors and on the implementation of various control measures and policies. Taking a broad perspective, we have shown that combustion processes in 2018 produced on average a lower amount of pollutants per unit of GHG than in the previous decades, with the important exception of ammonia emissions in transport. Comparing purposely developed indicators among countries at different development levels we have also shown that further improvement is in principle possible, if AP emissions per unit of GHG would align globally with the best observed values.

### Limitations of the study

As already mentioned in the introduction, the relative changes between GHG and AP have multiple causes, among which are the energy mix and the technological mix of the country, the effective deployment of appropriate end-of-pipe technologies, and the proper implementation of emissions control legislation. For this reason, it is useful to clearly stress that the goal of our article is not the detailed assessment of the various possible causes of GHG and AP changes, but instead we are interested in signaling areas, sectors, and pollutants for which the combined effect of all these factors causes AP and GHG behave or not in a synergistic way and to suggest possible policy and technological interpretations, that could and should better deepened in the future.

In other words, the main goal of our study consists of issuing a certain number of “warnings” at a macro level, summarized in the previous paragraphs, worth to be further investigated both from the technological and policy perspectives.

Clearly, an additional limitation of our study lies in restricting its scope to the combustion related emissions, largely consisting in CO_2_, and covering the about two-thirds of total global GHG emissions. Although we are well aware of the importance of other sectors and substances, their investigation would need the elaboration of other and purposely tuned indicators, currently outside the scope of our analysis.

## STAR★Methods

### Key resources table

**Table:** 

REAGENT or RESOURCE	SOURCE	IDENTIFIER
**Data**		

EDGARv6.0 provides emissions of the three main greenhouse gases (CO2, CH4, N2O) and fluorinated gases per sector and country.	Monforti Ferrario, Fabio; Crippa, Monica; Guizzardi, Diego; Muntean, Marilena; Schaaf, Edwin; Lo Vullo, Eleonora; Solazzo, Efisio; Olivier, Jos; Vignati, Elisabetta (2021): EDGAR v6.0 Greenhouse Gas Emissions. European Commission, Joint Research Center (JRC) [Dataset] PID: http://data.europa.eu/89h/97a67d67-c62e-4826-b873-9d972c4f670b	https://edgar.jrc.ec.europa.eu/dataset_ghg60
EDGARv6.1 provides emissions per sector and country for the following air pollutants: Ozone precursor gases: Carbon Monoxide (CO), Nitrogen Oxides (NOx), Non-Methane Volatile Organic Compounds (NMVOC) and Methane (CH4) Acidifying gases: Ammonia (NH3), Nitrogen oxides (NOx) and Sulfur Dioxide (SO2) Primary particulates: Fine Particulate Matter (PM10 and PM2.5) and Carbonaceous speciation (BC, OC)	Crippa, M., Guizzardi, D., Muntean, M., Schaaf, E., Monforti-Ferrario, F., Banja, M., Pagani, F. and Solazzo, E., EDGAR v6.1 global air pollutant emissions, European Commission, 2022, JRC129555.	https://edgar.jrc.ec.europa.eu/index.php/dataset_ap61

### Resources availability

#### Lead contact

Further information and requests should be directed to and will be fulfilled by the lead contact, Fabio Monforti-Ferrario (Fabio.Monforti-Ferrario@ec.europa.eu).

#### Data and code availability

EDGAR data used in this paper are freely available and downloadable on:

https://edgar.jrc.ec.europa.eu/dataset_ghg60 (GHG data).

https://edgar.jrc.ec.europa.eu/index.php/dataset_ap61 (Air Pollutants data).

### Methods details

EDGAR emissions are computed using a technology based emission factor approach consistently applied for all world countries.

Emissions for each compound *x* for a country *C* (EM_C,x_) are calculated as the sum over sectors *i* of specific emissions obtained by multiplying country-specific activity data (AD_C,i_) with a set of additional factors representing: the mix of *j* technologies (TECH_C,i,j_) for each sector *i*, the abatement percentages of the *k* end-of-pipe measures for each technology *j* (EOP_C,i,j,k_), the country-specific emission factor of pollutant *x* (EF_C,i,j,x_) for each sector *i* and technology *j* and the relative reduction efficiency (RED_C,i,j,k,x_) of the uncontrolled emission by installed abatement measure *k*.

In formulas, in a given sector, related emissions EM_C,x_ of a compund *x* in country *C* are computed in EDGAR as

(Equation 1) $${\text{EM}}_{C,x}=\sum_{i,j,k}[{AD}_{C,i}\;{TECH}_{C,i,j}\;{EOP}_{C,i,j,k}\;{EF}_{C,i,j,x}\;\left(1-{RED}_{C,i,j,k,x}\;\right)]$$

Implied Emission Factors (IEF) are defined as emissions of a given sector divided by the total activity data of the same sector, usually expressed in units of emissions per energy consumed or fuel burnt.

More in detail, IEF_C,x_ for a compound *x* in country *C* is defined as

(Equation 2) $${\text{IEF}}_{C,x}={\text{EM}}_{C,x}\;/\sum_{i}{AD}_{C,i}=\sum_{i,j,k}[{AD}_{C,i}\;{TECH}_{C,i,j}\;{EOP}_{C,i,j,k}\;{EF}_{C,i,j,x}\;\left(1-{RED}_{C,i,j,k,x}\;\right)]/\;\sum_{i}{AD}_{C,i}$$

Considering this definitions, the ratio R_P/G,C_ between the emissions of an air pollutant *P* and a greenhouse gas *G* in a given country *C* can be then expressed as

(Equation 3) $$R_{P/G,C}={\text{EM}}_{C,P}/\;{\text{EM}}_{C,G}=\left({\text{EM}}_{C,P}/\sum_{i,f}{AD}_{C,i}\right)/\left(\sum_{i,f}{AD}_{C,i}/{\text{EM}}_{C,G}\right)={\text{IEF}}_{C,P}/\;{\text{IEF}}_{C,G}$$

In view of this identity, the trends for the different types of *R*_*P/G,C*_ presented in the results section can be easily interpreted as showing the relative changes of IEFs for air pollutants versus IEFs for GHG emissions. IEFs changes in time provide a view of how emissions have *overall* changed inside a given sector and country, taking into consideration e.g., fuel shifts and changes in subsectors relative weight.

#### Data sources

EDGAR depends on a number of sources of international statistics for the underlying data. Foremost among these is the International Energy Agency (IEA) for what concerns fossil fuels combustion. The IEA and the JRC are committed to the yearly co-production of consistent fossil CO_2_ emissions estimates up to the year t-1, directly using IEA CO_2_ emissions from fossil fuel combustion and JRC computations of CO_2_ process emissions. In the case of the sectors analysed in this study, activity data (AD_C,i_) are then mostly based on IEA World Energy Balances 2019.^10^
