# Supplementary material for: Addressing the different paces of climate and air quality combustion emissions across the world
Source: iScience. 2023 Dec 7;27(1):108686. doi: 10.1016/j.isci.2023.108686 (PMC10770704; doi:10.1016/j.isci.2023.108686)
Supplement: Document S1. Figures S1–S24 and Tables S1–S4 [file mmc1.pdf]

iScience, Volume *27*

Supplemental information

Addressing the different paces of climate and air quality combustion emissions across the world

Fabio Monforti-Ferrario, Monica Crippa, and Enrico Pisoni

The file includes:

Appendix
Tables S1 to S4
Figs. S1 to S24

Appendix.
Former Annex I (FAI) countries

Australia
Austria
Belarus
Belgium
Bulgaria
Canada
Croatia
Czech Republic
Denmark
European Union
Estonia
Finland
France
Germany
Greece
Hungary
Iceland
Ireland
Italy
Japan
Latvia
Liechtenstein
Lithuania
Luxembourg
Monaco
Netherlands
New Zealand
Norway
Poland
Portugal
Romania
Russian Federation
Slovak Republic
Slovenia
Spain
Sweden
Switzerland
Turkey
Ukraine
United Kingdom of Great Britain and Northern Ireland
United States of America

Former Non Annex I (FNAI) countries
All other countries

$PM_{2.5}$	EU27	China	FAI	FNAI	World
**1.A**	0.32	0.96	-0.65	0.98	0.97
**1.A.1**	-0.31	0.97	-0.79	0.98	0.91
**1.A.2**	0.98	0.99	0.97	1.00	0.99
**1.A.3**	0.27	0.94	-0.36	0.94	0.98
**1.A.4**	0.68	0.83	0.87	0.96	0.91

Table S.1: Pearson’s correlation coefficient for $PM_{2.5}$ and CO_2 in the 1970-2018 for selected IPCC sectors and world regions. Related to Tables 6-9.

NO_x	EU27	China	FAI	FNAI	World
**1.A**	0.563	0.990	-0.117	0.996	0.960
**1.A.1**	0.017	0.975	0.197	0.992	0.988
**1.A.2**	0.997	0.996	0.997	0.998	0.995
**1.A.3**	-0.448	0.958	-0.561	0.969	0.669
**1.A.4**	0.926	0.651	0.984	0.994	0.977

Table S.2: Pearson’s correlation coefficient for NO_x and CO_2 in the 1970-2018 for selected IPCC sectors and world regions. Related to Tables 6-9.

SO_2	EU27	China	FAI	FNAI	World
**1.A**	0.35	0.92	-0.61	0.97	-0.35
**1.A.1**	-0.23	0.60	-0.63	0.91	-0.33
**1.A.2**	0.98	0.99	0.98	0.99	0.80
**1.A.3**	-0.95	0.97	-0.97	0.93	0.86
**1.A.4**	0.74	0.63	0.89	0.02	-0.89

Table S.3: Pearson’s correlation coefficient for SO_2 and CO_2 in the 1970-2018 for selected IPCC sectors and world regions. Related to Tables 6-9.

NH_3	EU27	China	FAI	FNAI	World
**1.A.3**	0.94	0.99	0.90	0.99	0.95

Table S.4: Pearson’s correlation coefficient for NH_3 and CO_2 in the 1970-2018 in the 1.A.3 IPCC sector in selected world regions. Related to Tables 6-9.

Figures S.1 to S.24: Top panel: Time series (1970-2018) of selected indicators for the same IPCC sector and regions listed in Tables 2 to 5. For improved readability Y-axis is in logarithmic scale. Bottom left: CO_2 emissions (1970-2018) for the same IPCC sector and regions listed in Tables 2 to 5. Bottom right: emissions (1970-2018) of selected pollutants for the same IPCC sector and regions listed in Tables 2 to 5. Related to Figure 1.

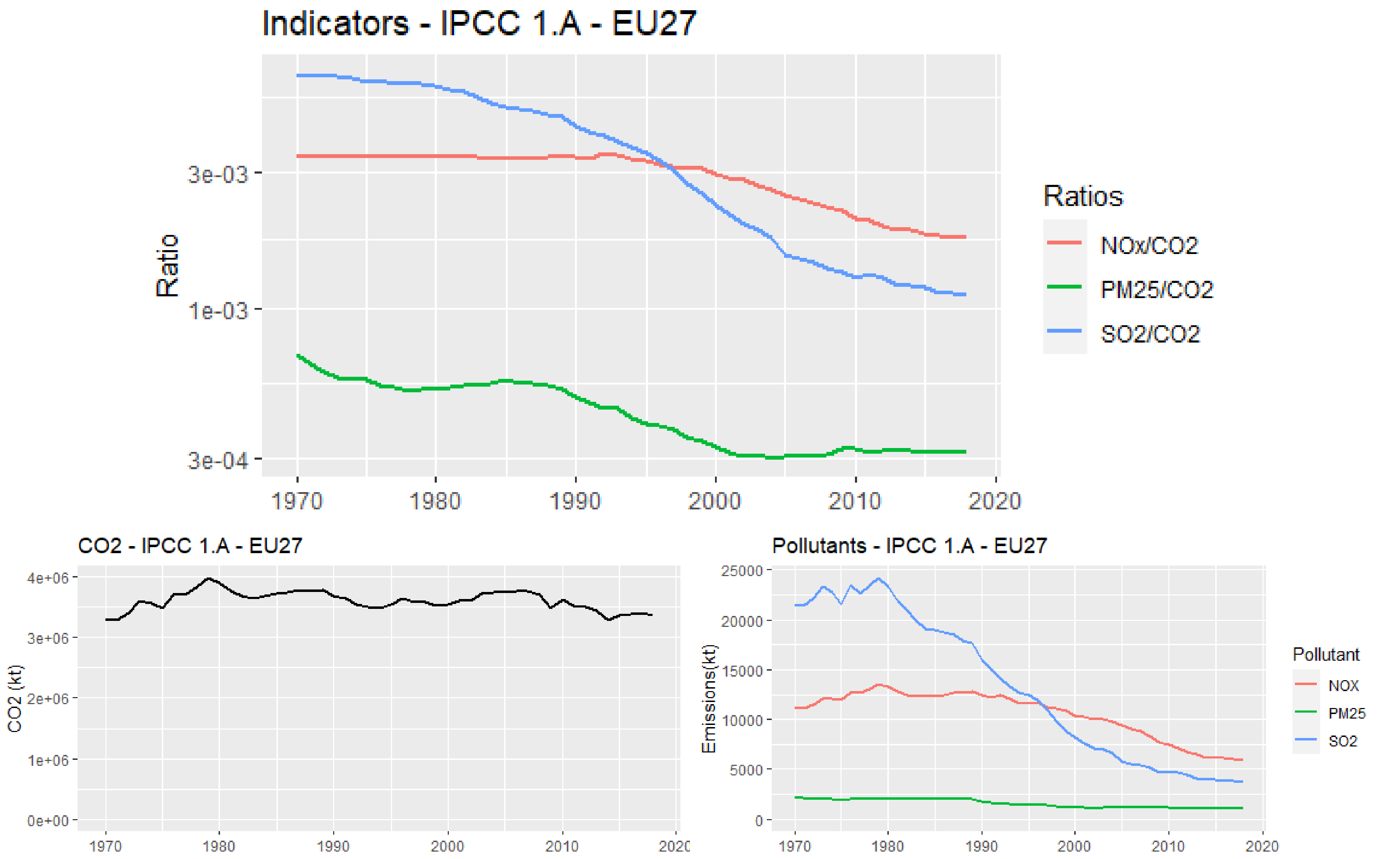

Figure S.1 – Top panel: Time series (1970-2018) of selected indicators in the IPCC 1.A (Combustion) sector in EU27. Bottom left: CO_2 emissions (1970-2018) in 1.A sector in EU27. Bottom right: emissions (1970-2018) of selected pollutants in 1.A sector in EU27. Related to Figure 1.

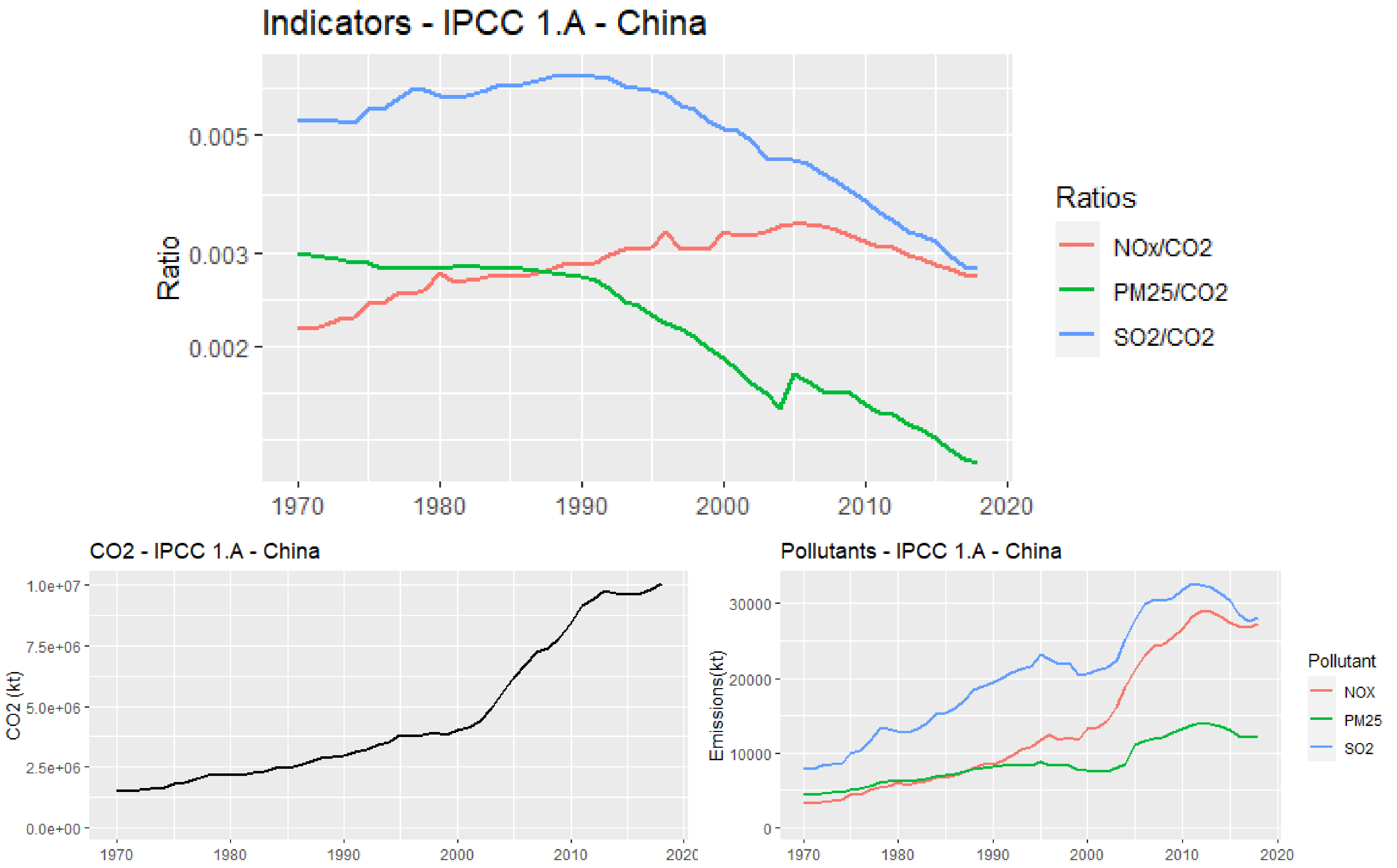

Figure S.2 – Top panel: Time series (1970-2018) of selected indicators in the IPCC 1.A (Combustion) sector in China. Bottom left: CO_2 emissions (1970-2018) in 1.A sector in China. Bottom right: emissions (1970-2018) of selected pollutants in 1.A sector in China. Related to Figure 1.

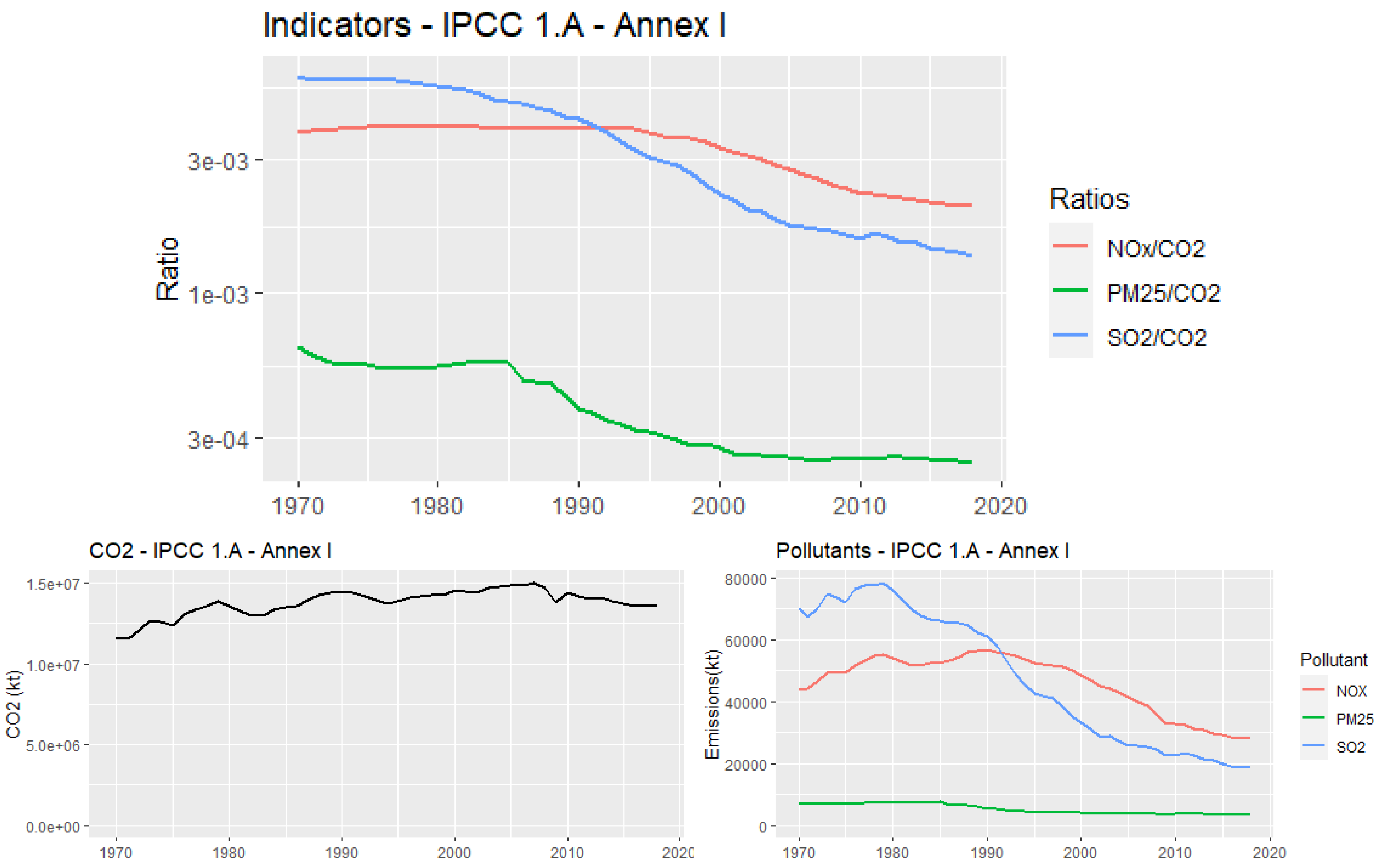

Figure S.3 – Top panel: Time series (1970-2018) of selected indicators in the IPCC 1.A (Combustion) sector in FAI countries. Bottom left: CO_2 emissions (1970-2018) in 1.A sector in FAI countries. Bottom right: emissions (1970-2018) of selected pollutants in 1.A sector in FAI countries. Related to Figure 1.

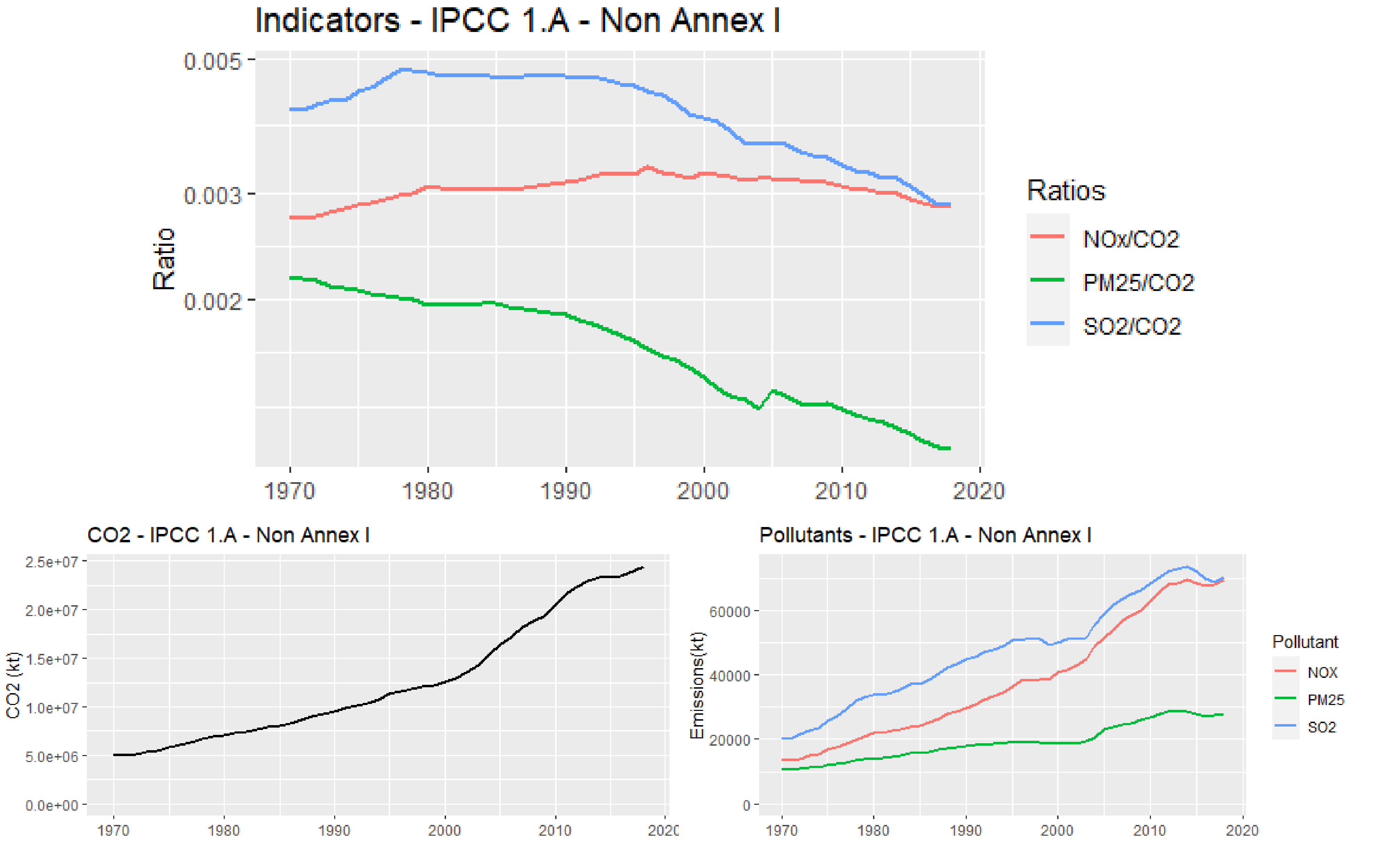

Figure S.4 – Top panel: Time series (1970-2018) of selected indicators in the IPCC 1.A (Combustion) sector in FNAI countries. Bottom left: CO_2 emissions (1970-2018) in 1.A sector in FNAI countries. Bottom right: emissions (1970-2018) of selected pollutants in 1.A sector in FNAI countries. Related to Figure 1.

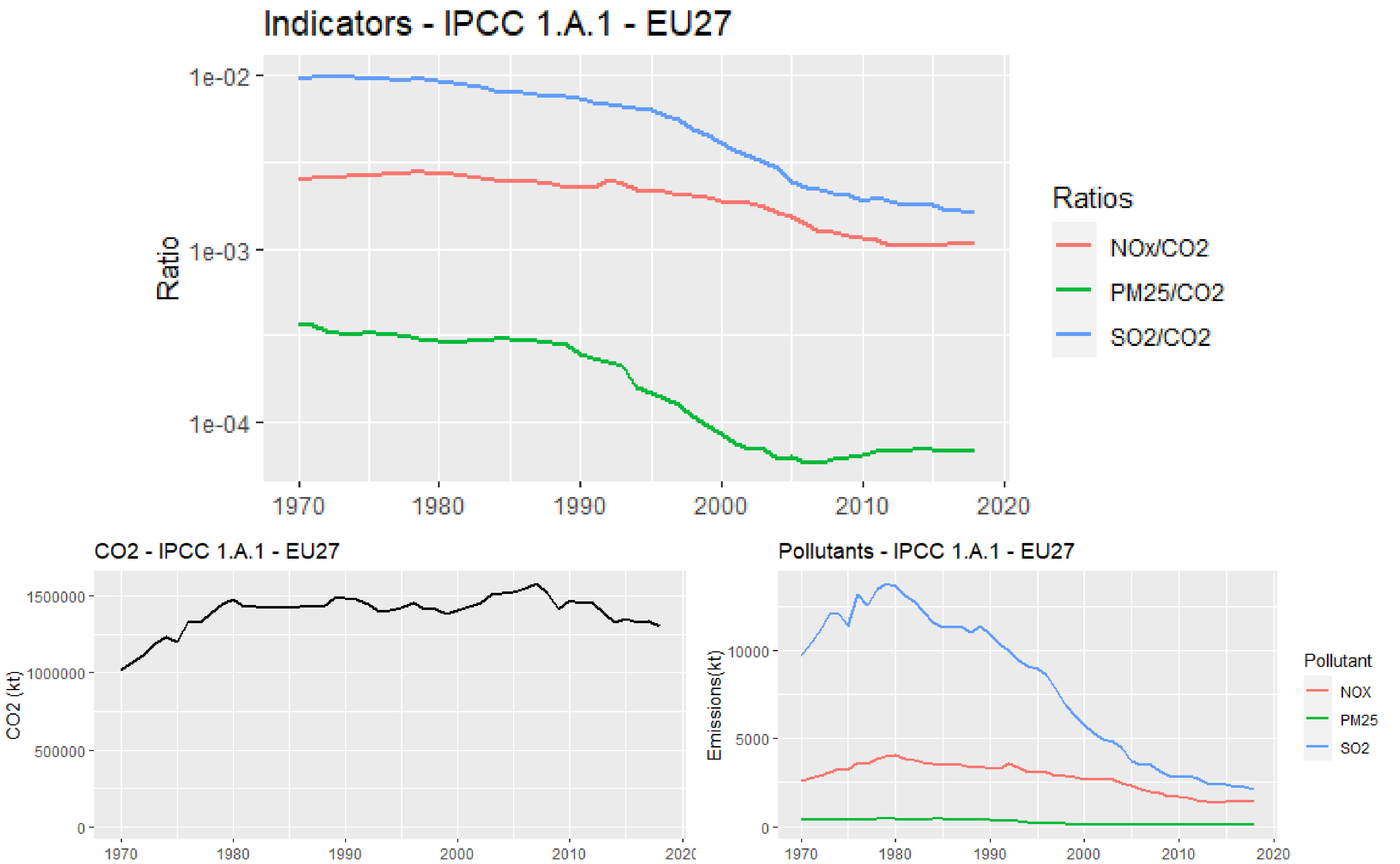

Figure S.5 – Top panel: Time series (1970-2018) of selected indicators in the IPCC 1.A.1 (Energy Industry) sector in EU27. Bottom left: CO_2 emissions (1970-2018) in 1.A.1 sector in EU27. Bottom right: emissions (1970-2018) of selected pollutants in 1.A.1 sector in EU27. Related to Figure 1.

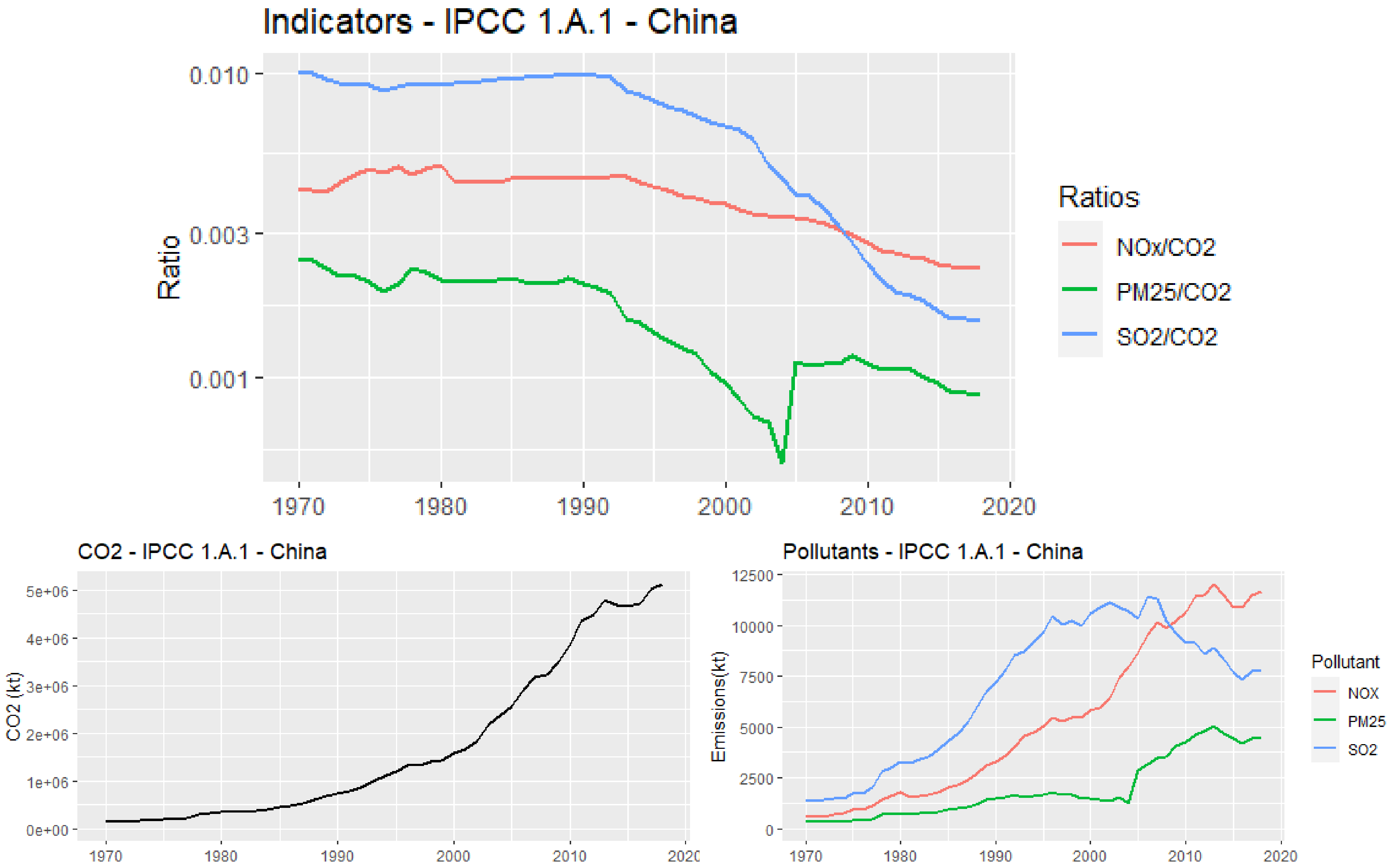

Figure S.6 – Top panel: Time series (1970-2018) of selected indicators in the IPCC 1.A.1 (Energy Industry) sector in China. Bottom left: CO_2 emissions (1970-2018) in 1.A.1 sector in China. Bottom right: emissions (1970-2018) of selected pollutants in 1.A.1 sector in China. Related to Figure 1.

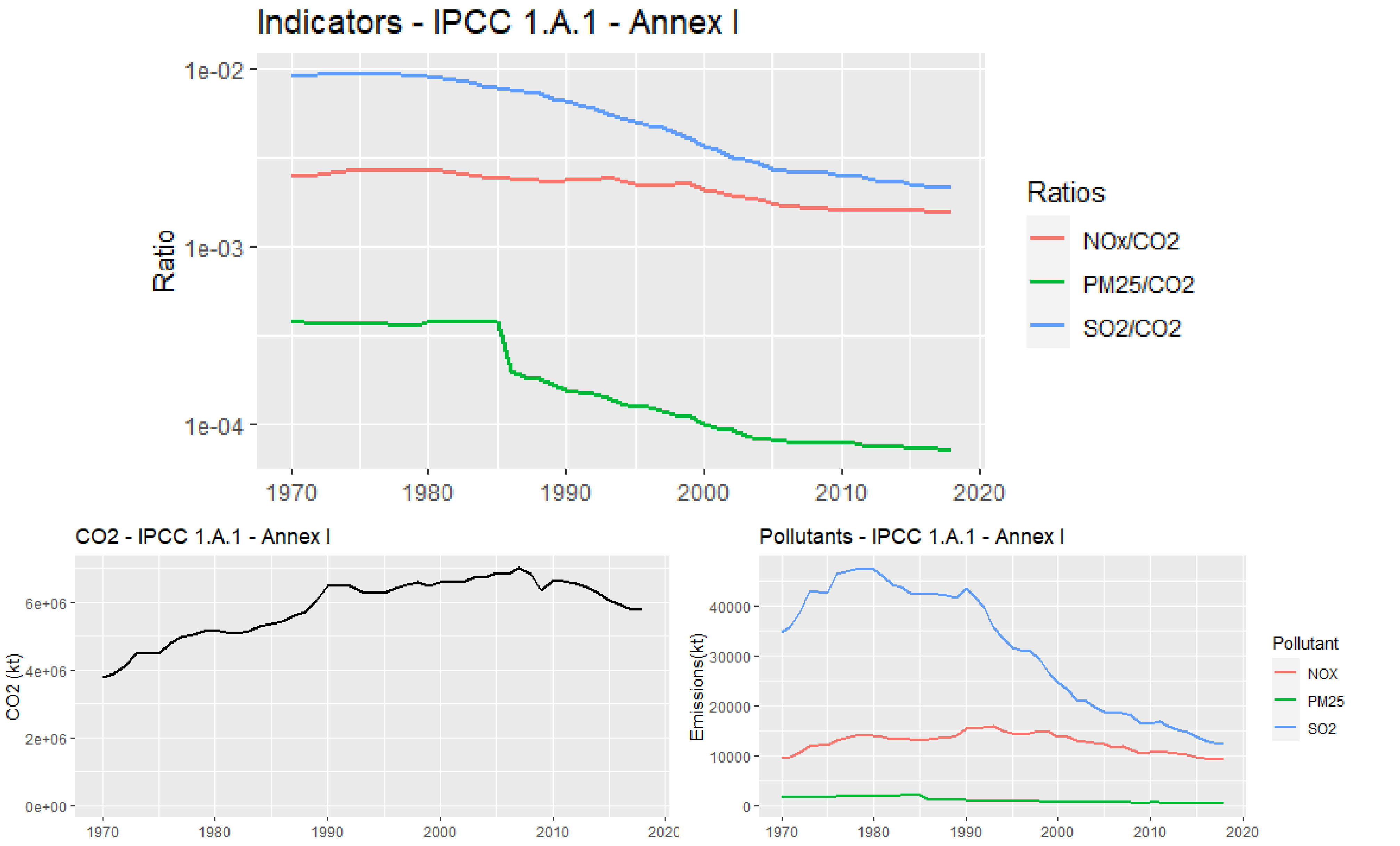

Figure S.7 – Top panel: Time series (1970-2018) of selected indicators in the IPCC 1.A.1 (Energy Industry) sector in FAI countries. Bottom left: CO_2 emissions (1970-2018) in 1.A.1 sector in FAI countries. Bottom right: emissions (1970-2018) of selected pollutants in 1.A.1 sector in FAI countries. Related to Figure 1.

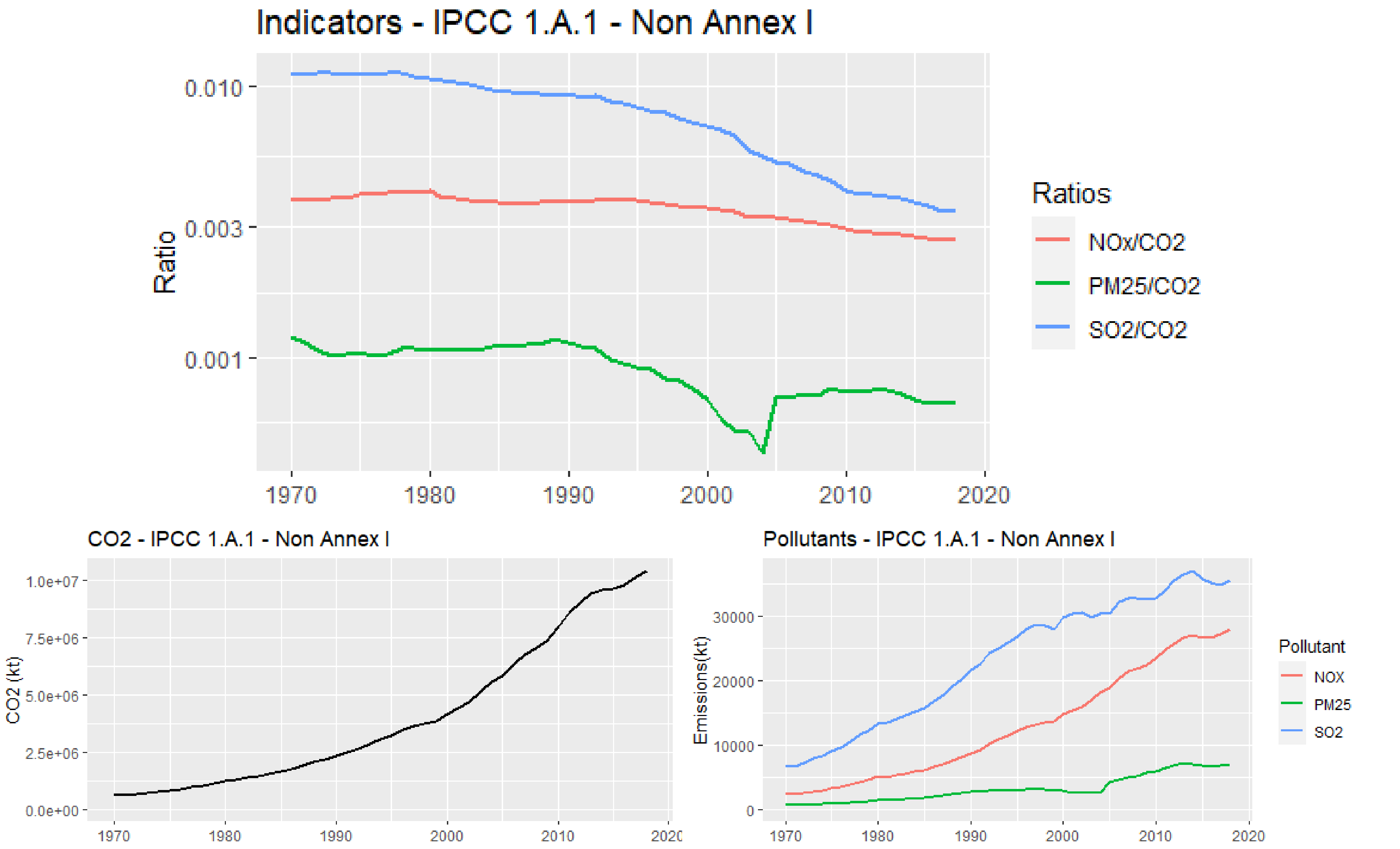

Figure S.8 – Top panel: Time series (1970-2018) of selected indicators in the IPCC 1.A.1 (Energy Industry) sector in FNAI countries. Bottom left: CO_2 emissions (1970-2018) in 1.A.1 sector in FNAI countries. Bottom right: emissions (1970-2018) of selected pollutants in 1.A.1 sector in FNAI countries. Related to Figure 1.

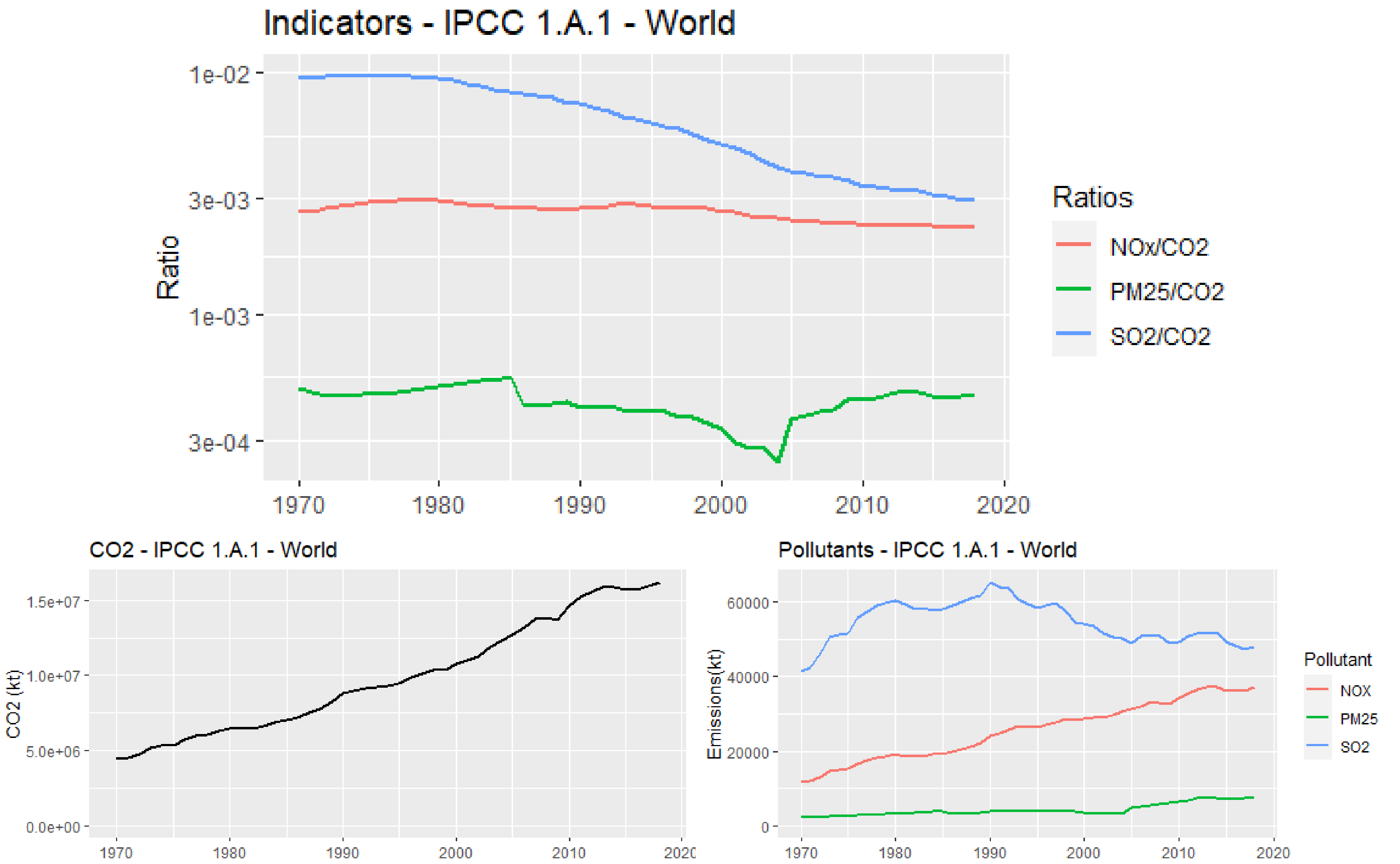

Figure S.9 – Top panel: Time series (1970-2018) of selected indicators in the IPCC 1.A.1 (Energy Industry) sector in all world countries. Bottom left: CO_2 emissions (1970-2018) in 1.A.1 sector in all world countries. Bottom right: emissions (1970-2018) of selected pollutants in 1.A.1 sector in all world countries. Related to Figure 1.

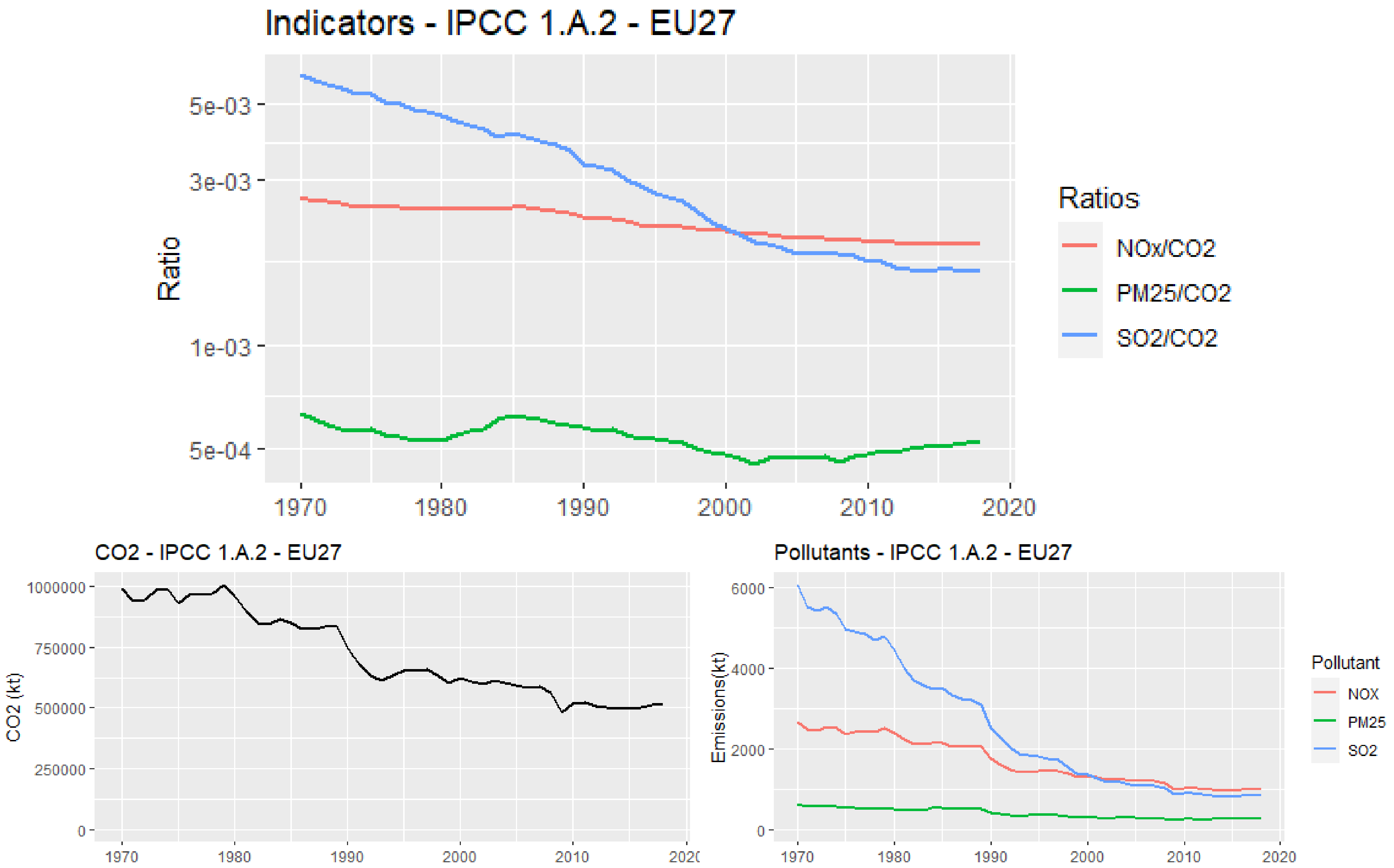

Figure S.10 – Top panel: Time series (1970-2018) of selected indicators in the IPCC 1.A.2 (Manufacturing and construction) sector in EU27. Bottom left: CO_2 emissions (1970-2018) in 1.A.2 sector in EU27. Bottom right: emissions (1970-2018) of selected pollutants in 1.A.2 sector in EU27. Related to Figure 1.

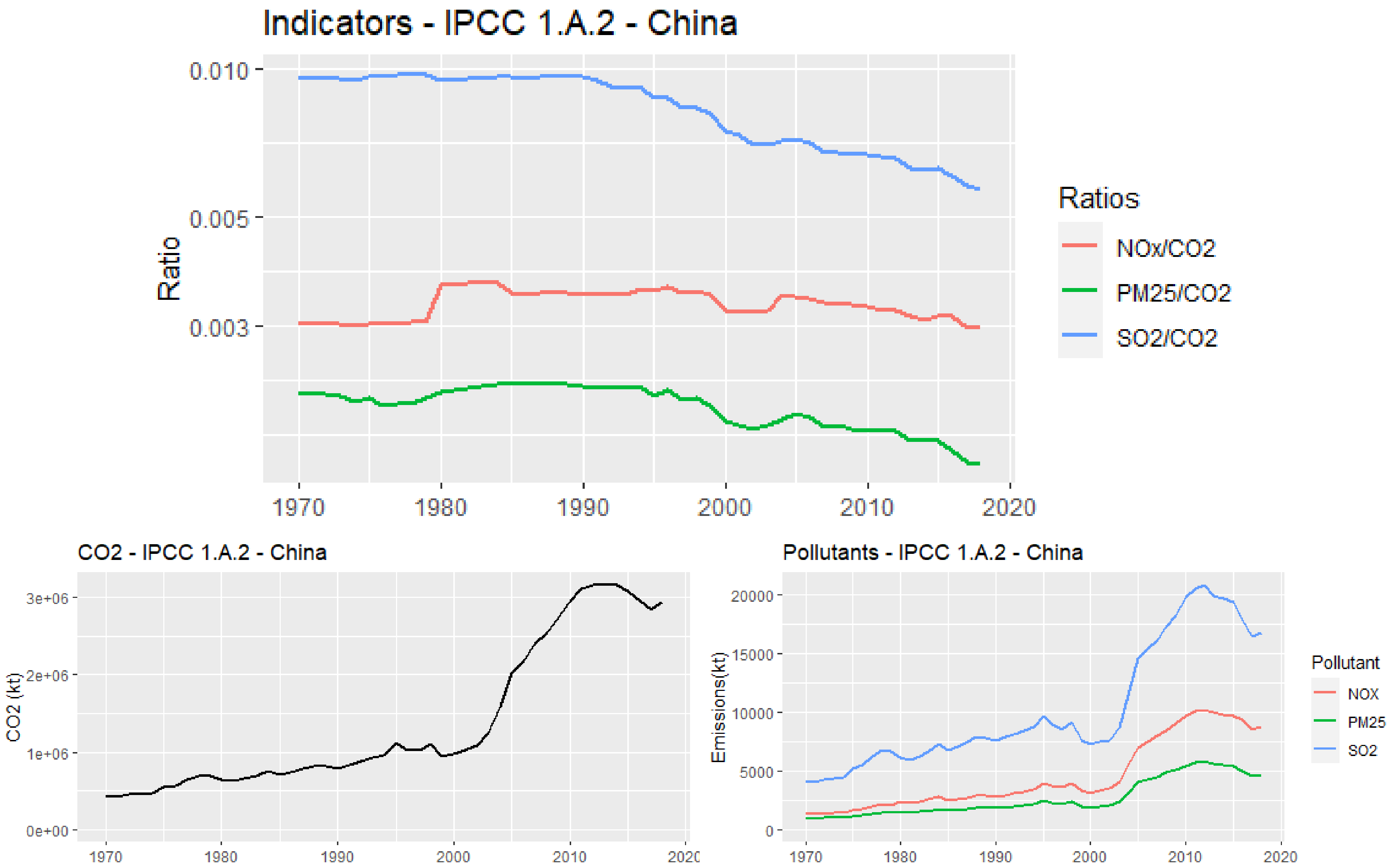

Figure S.11 – Top panel: Time series (1970-2018) of selected indicators in the IPCC 1.A.2 (Manufacturing and construction) sector in China. Bottom left: CO_2 emissions (1970-2018) in 1.A.2 sector in China. Bottom right: emissions (1970-2018) of selected pollutants in 1.A.2 sector in China. Related to Figure 1.

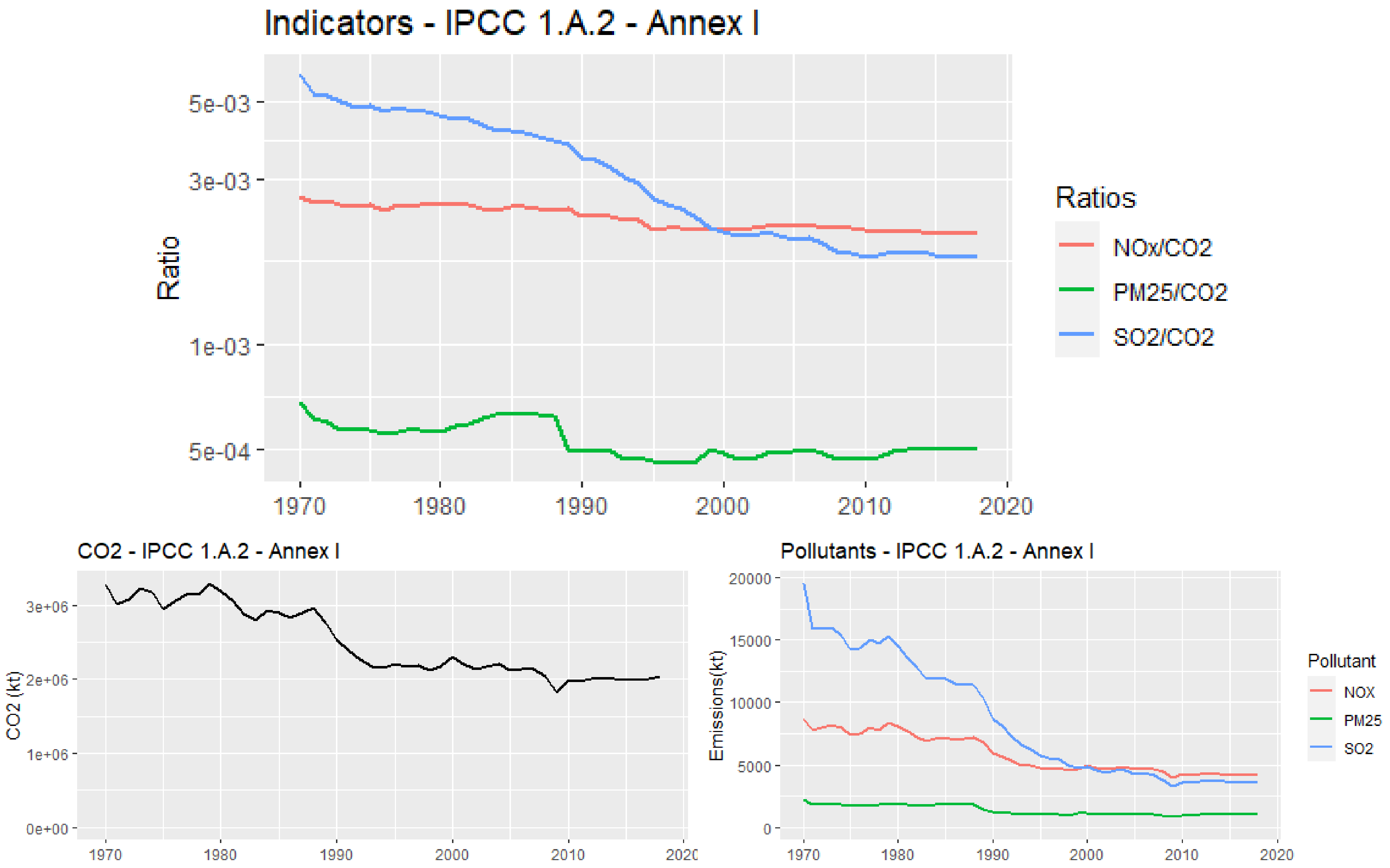

Figure S.12 – Top panel: Time series (1970-2018) of selected indicators in the IPCC 1.A.2 (Manufacturing and construction) sector in FAI countries. Bottom left: CO_2 emissions (1970-2018) in 1.A.2 sector in FAI countries. Bottom right: emissions (1970-2018) of selected pollutants in 1.A.2 sector in FAI countries. Related to Figure 1.

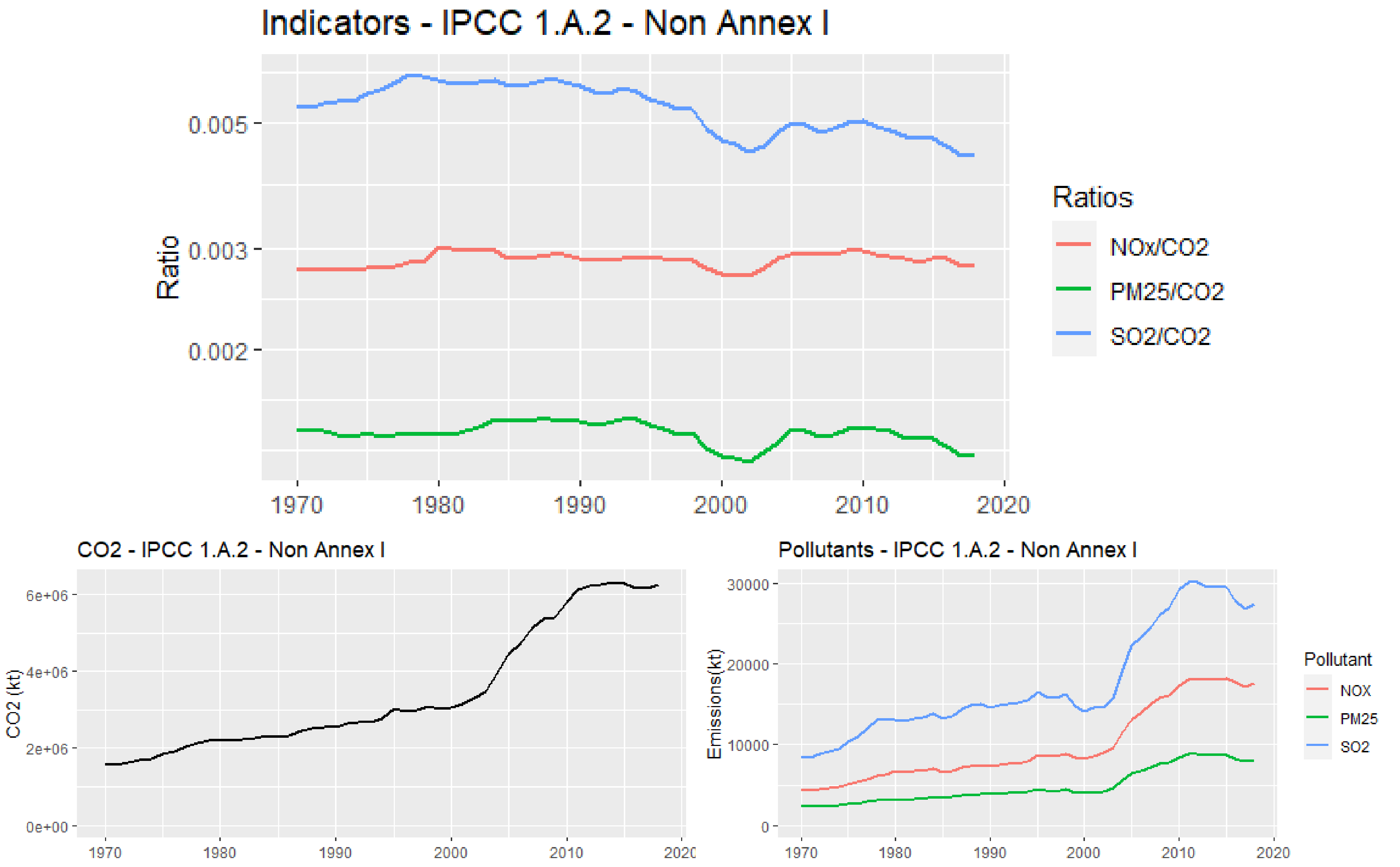

Figure S.13 – Top panel: Time series (1970-2018) of selected indicators in the IPCC 1.A.2 (Manufacturing and construction) sector in FNAI countries. Bottom left: CO_2 emissions (1970-2018) in 1.A.2 sector in FNAI countries. Bottom right: emissions (1970-2018) of selected pollutants in 1.A.2 sector in FNAI countries. Related to Figure 1.

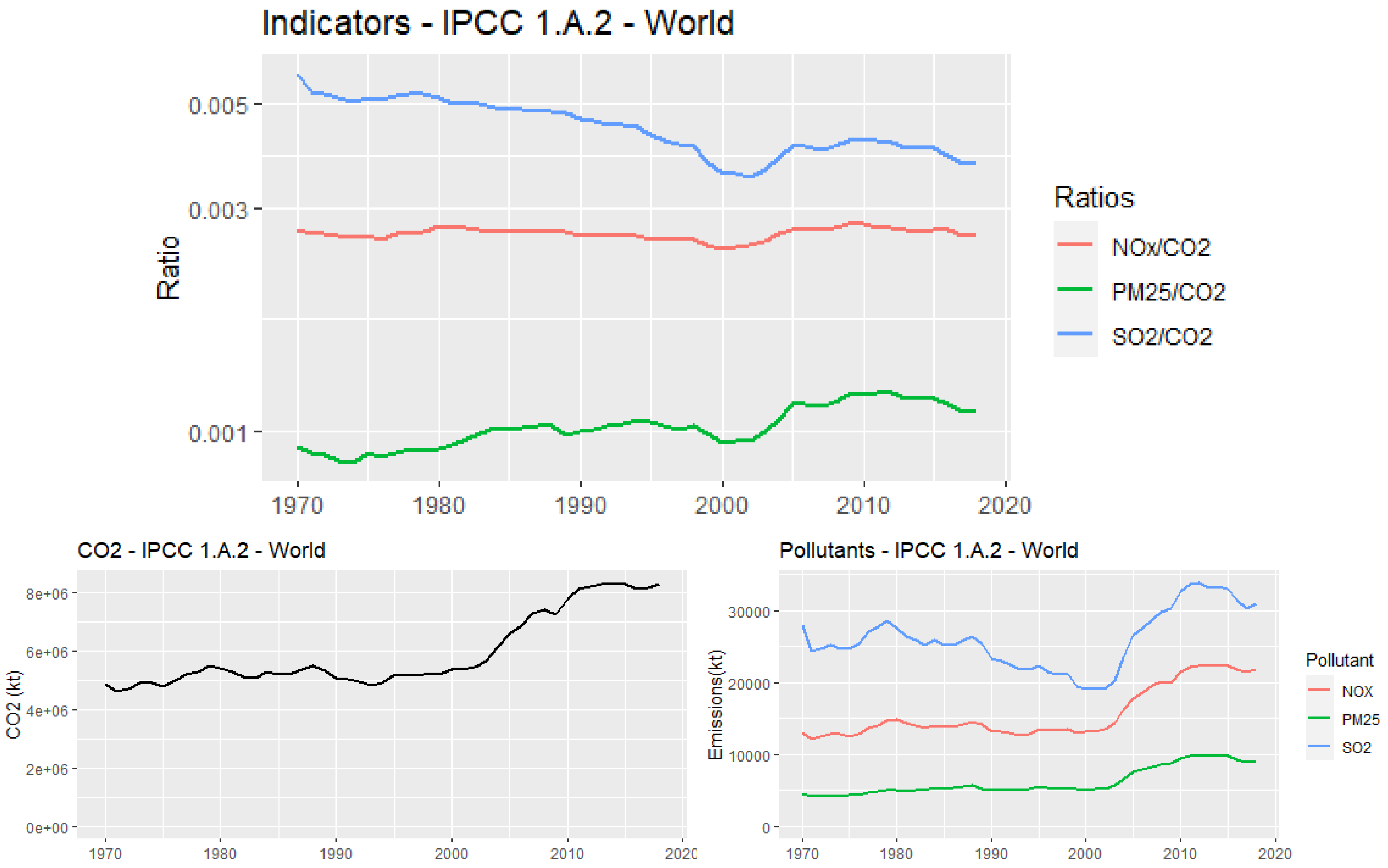

Figure S.14 – Top panel: Time series (1970-2018) of selected indicators in the IPCC 1.A.2 (Manufacturing and construction) sector in all world countries. Bottom left: CO_2 emissions (1970-2018) in 1.A.2 sector in all world countries. Bottom right: emissions (1970-2018) of selected pollutants in 1.A.2 sector in all world countries. Related to Figure 1.

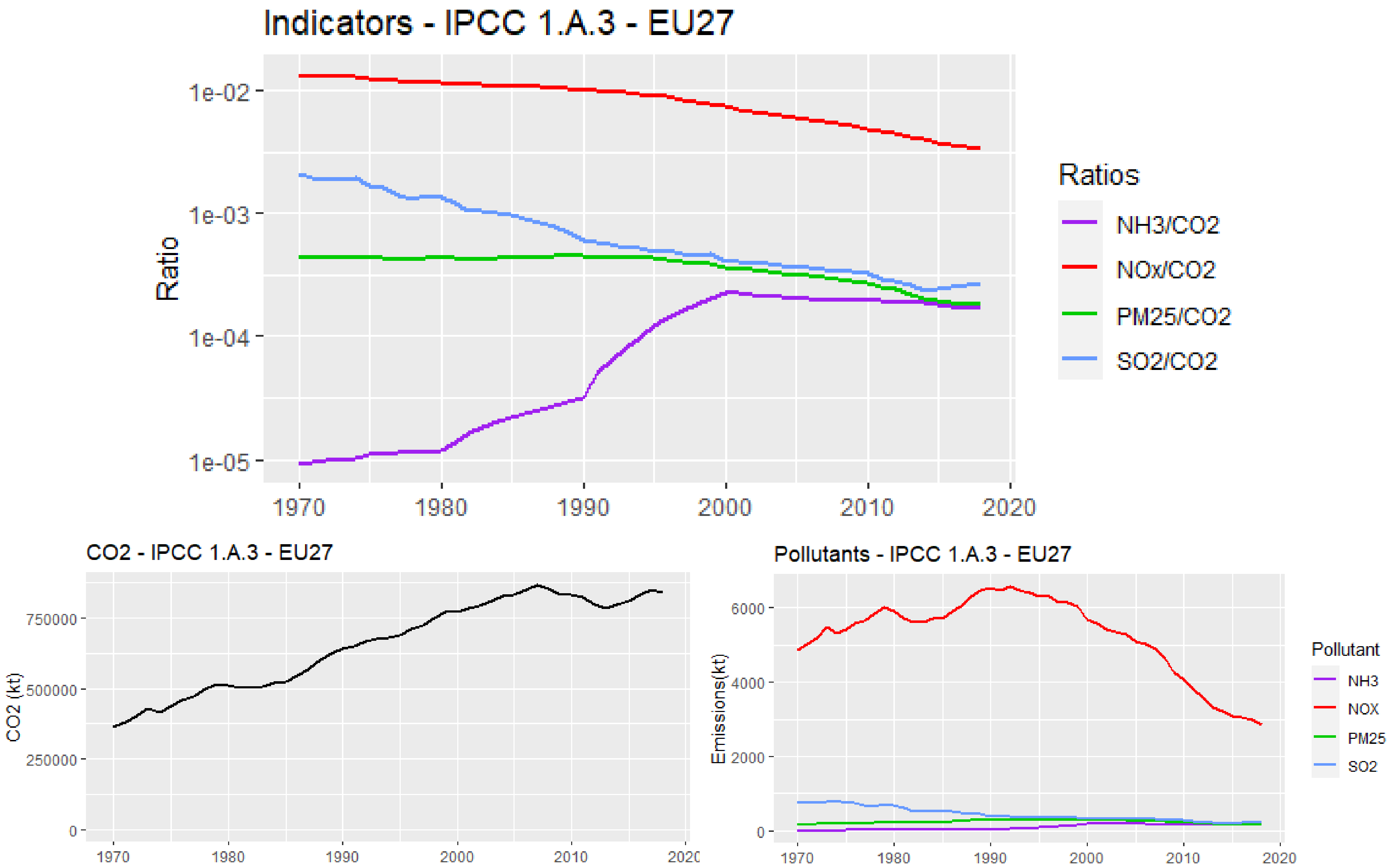

Figure S.15 – Top panel: Time series (1970-2018) of selected indicators in the IPCC 1.A.3 (Transport) sector in EU27. Bottom left: CO_2 emissions (1970-2018) in 1.A.3 sector in EU27. Bottom right: emissions (1970-2018) of selected pollutants in 1.A.3 sector in EU27. Related to Figure 1.

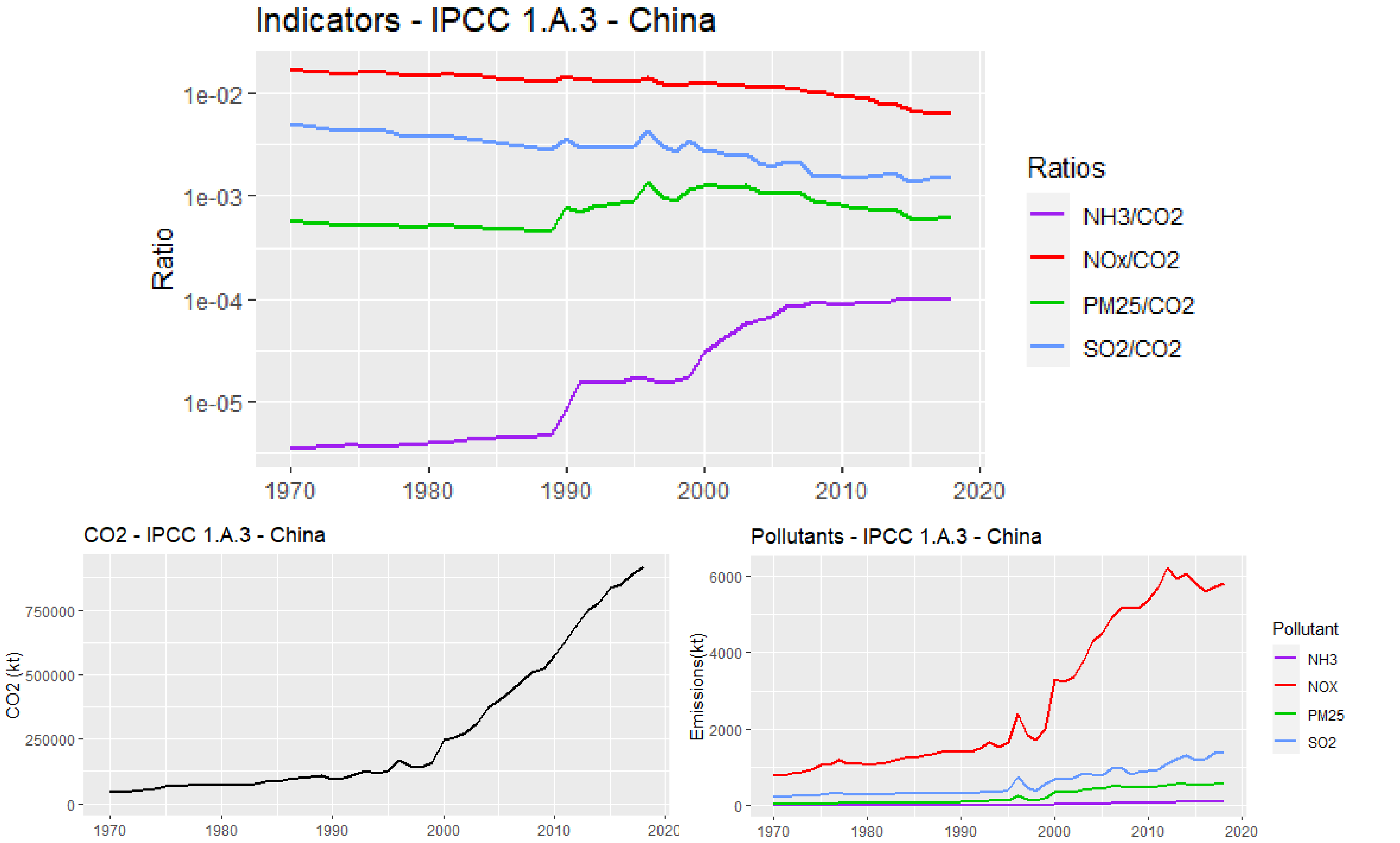

Figure S.16 – Top panel: Time series (1970-2018) of selected indicators in the IPCC 1.A.3 (Transport) sector in China. Bottom left: CO_2 emissions (1970-2018) in 1.A.3 sector in China. Bottom right: emissions (1970-2018) of selected pollutants in 1.A.3 sector in China. Related to Figure 1.

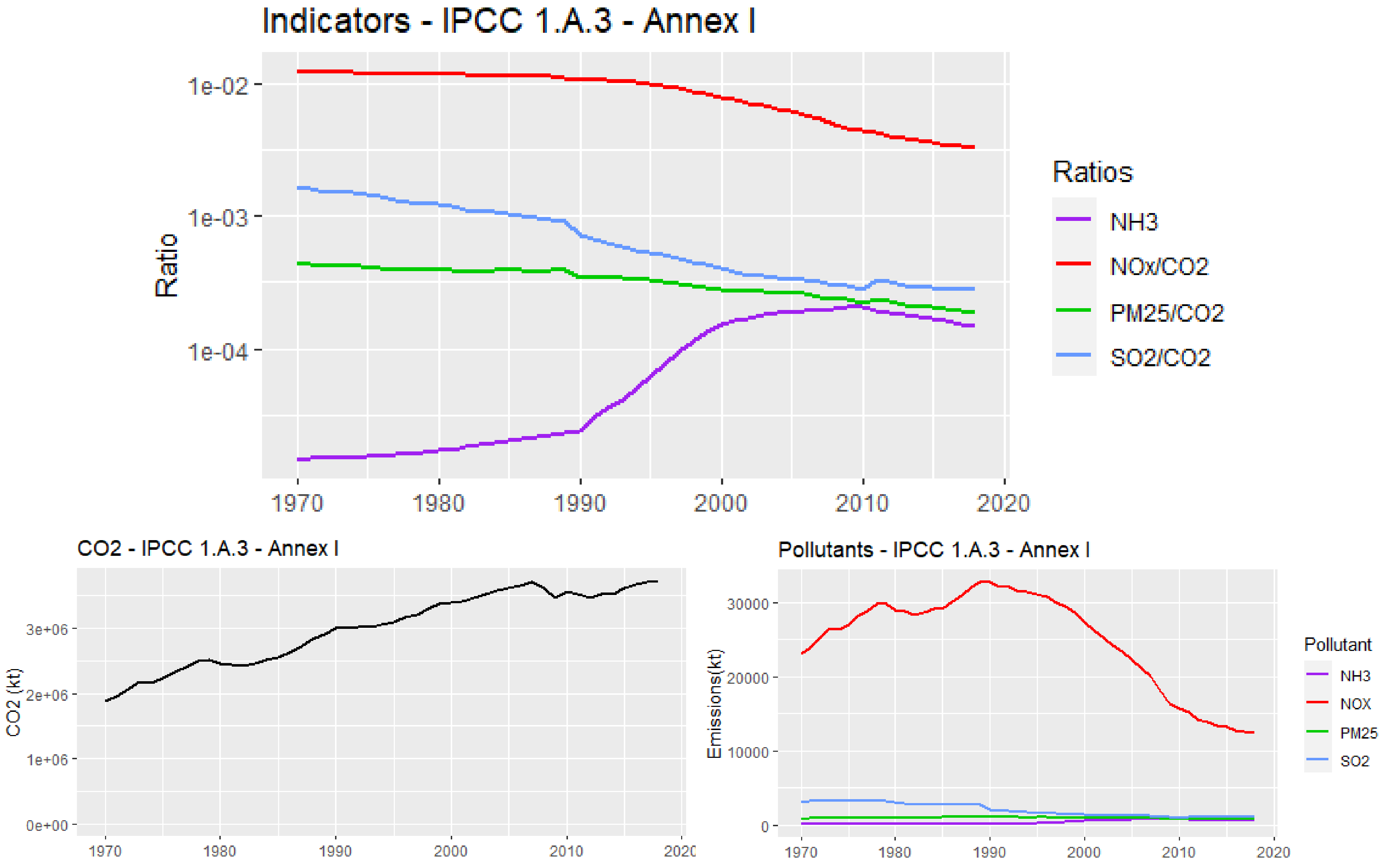

Figure S.17 – Top panel: Time series (1970-2018) of selected indicators in the IPCC 1.A.3 (Transport) sector in FAI countries. Bottom left: CO_2 emissions (1970-2018) in 1.A.3 sector in FAI countries. Bottom right: emissions (1970-2018) of selected pollutants in 1.A.3 sector in FAI countries. Related to Figure 1.

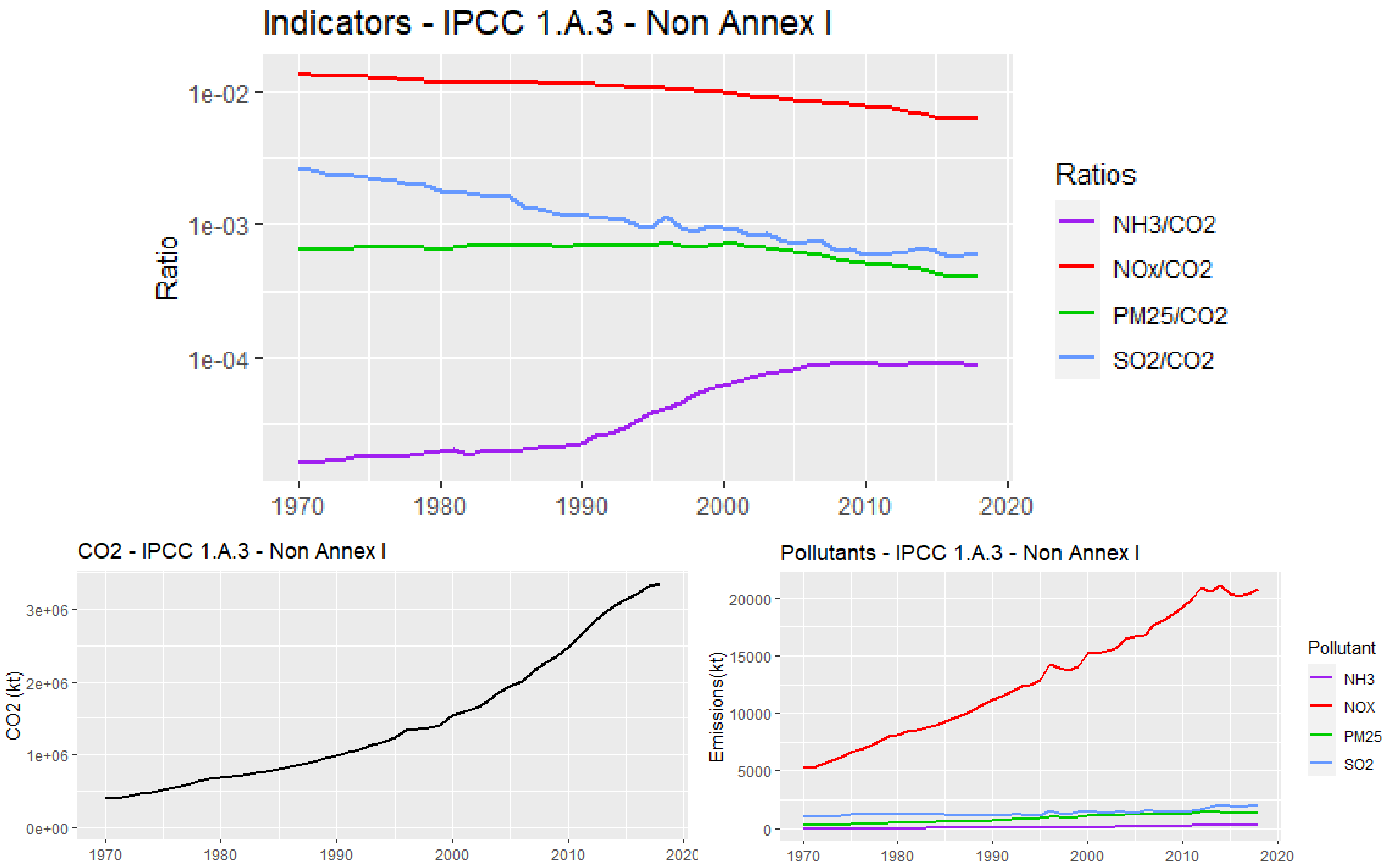

Figure S.18 – Top panel: Time series (1970-2018) of selected indicators in the IPCC 1.A.3 (Transport) sector in FNAI countries. Bottom left: CO_2 emissions (1970-2018) in 1.A.3 sector in FNAI countries. Bottom right: emissions (1970-2018) of selected pollutants in 1.A.3 sector in FNAI countries. Related to Figure 1.

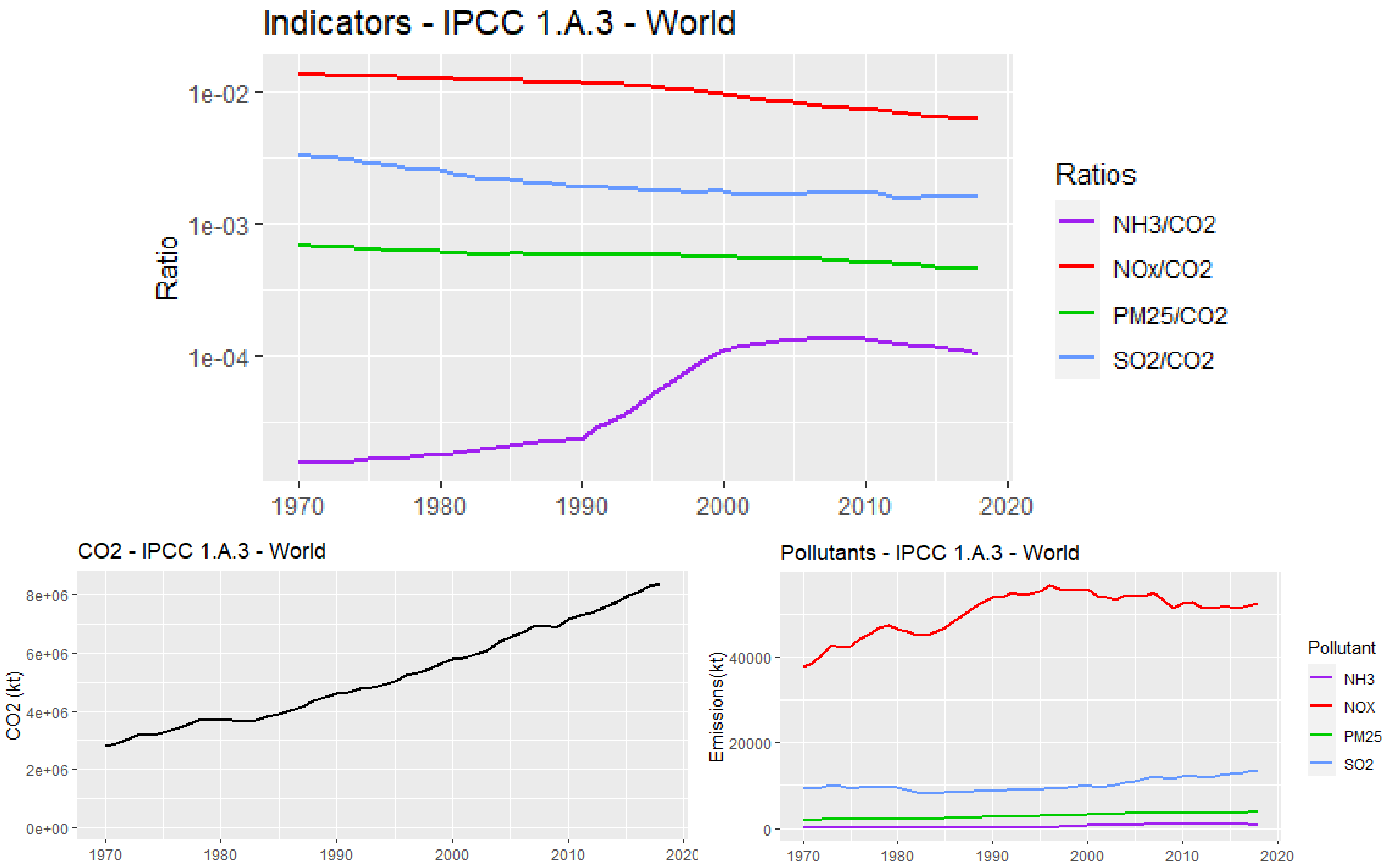

Figure S.19 – Top panel: Time series (1970-2018) of selected indicators in the IPCC 1.A.3 (Transport) sector in all world countries. Bottom left: CO_2 emissions (1970-2018) in 1.A.3 sector in all world countries. Bottom right: emissions (1970-2018) of selected pollutants in 1.A.3 sector in all world countries. Related to Figure 1.

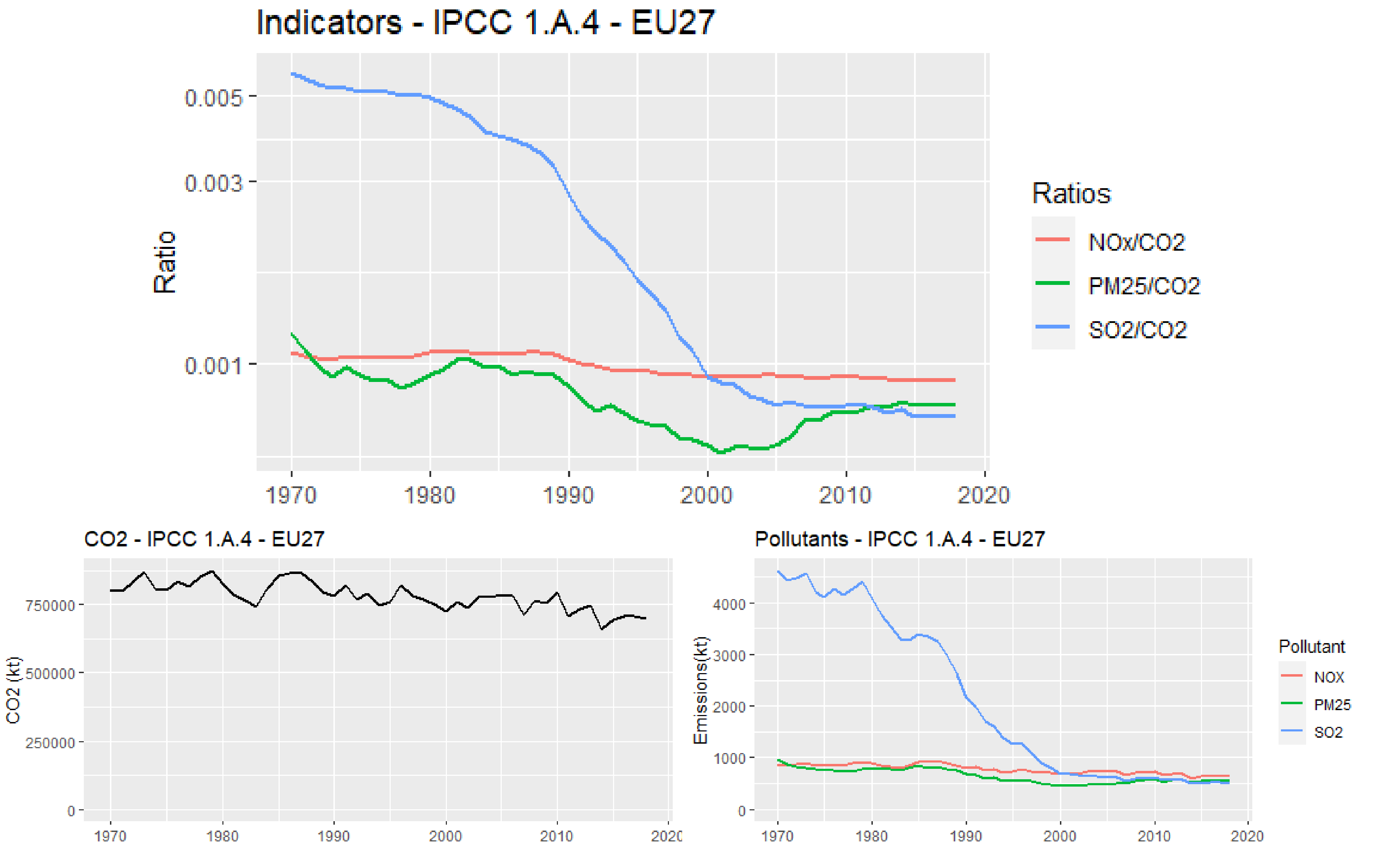

Figure S.20 – Top panel: Time series (1970-2018) of selected indicators in the IPCC 1.A.4 (Buildings) sector in EU27. Bottom left: CO_2 emissions (1970-2018) in 1.A.4 sector in EU27. Bottom right: emissions (1970-2018) of selected pollutants in 1.A.4 sector in EU27. Related to Figure 1.

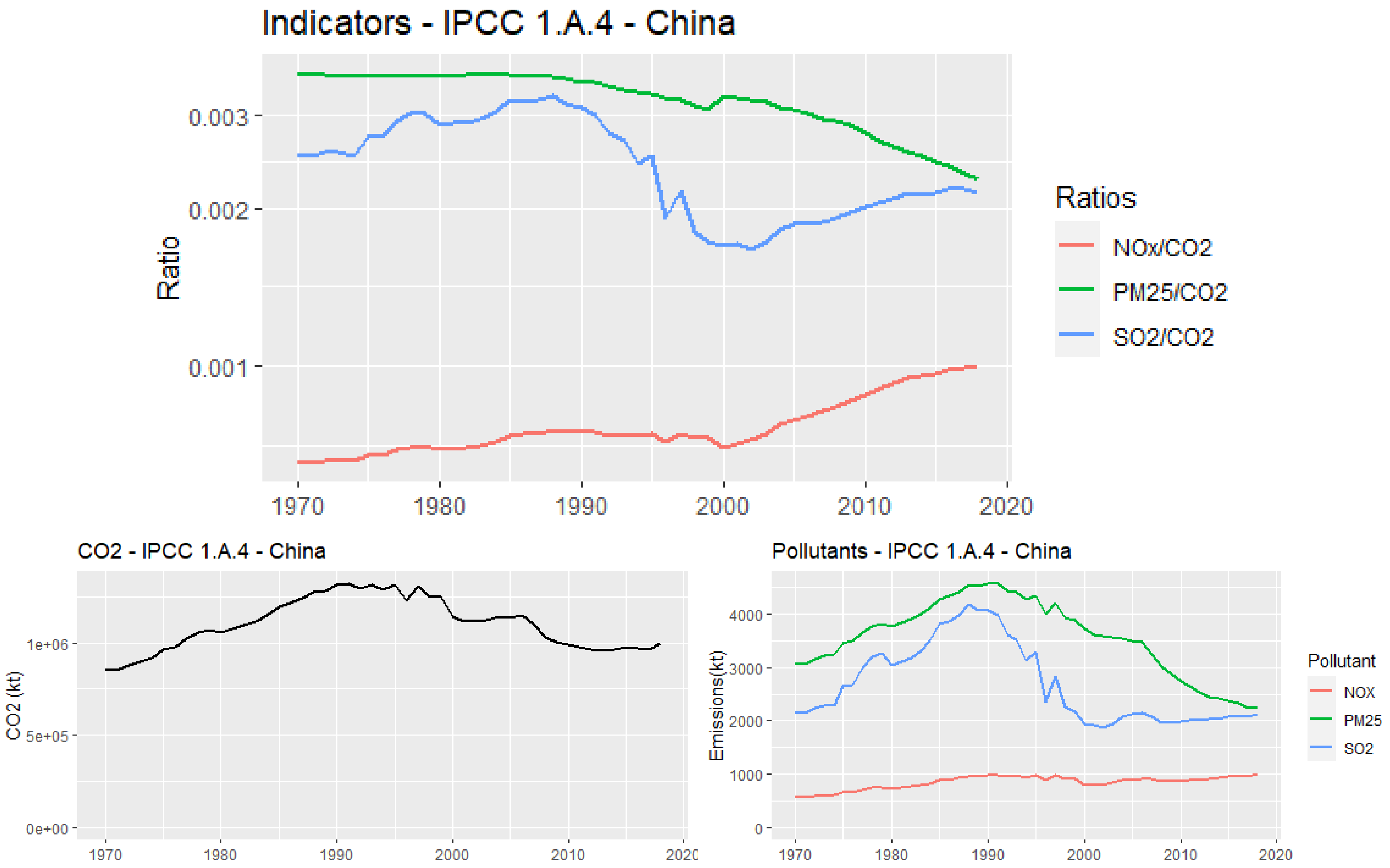

Figure S.21 – Top panel: Time series (1970-2018) of selected indicators in the IPCC 1.A.4 (Buildings) sector in China. Bottom left: CO_2 emissions (1970-2018) in 1.A.4 sector in China. Bottom right: emissions (1970-2018) of selected pollutants in 1.A.4 sector in China. Related to Figure 1.

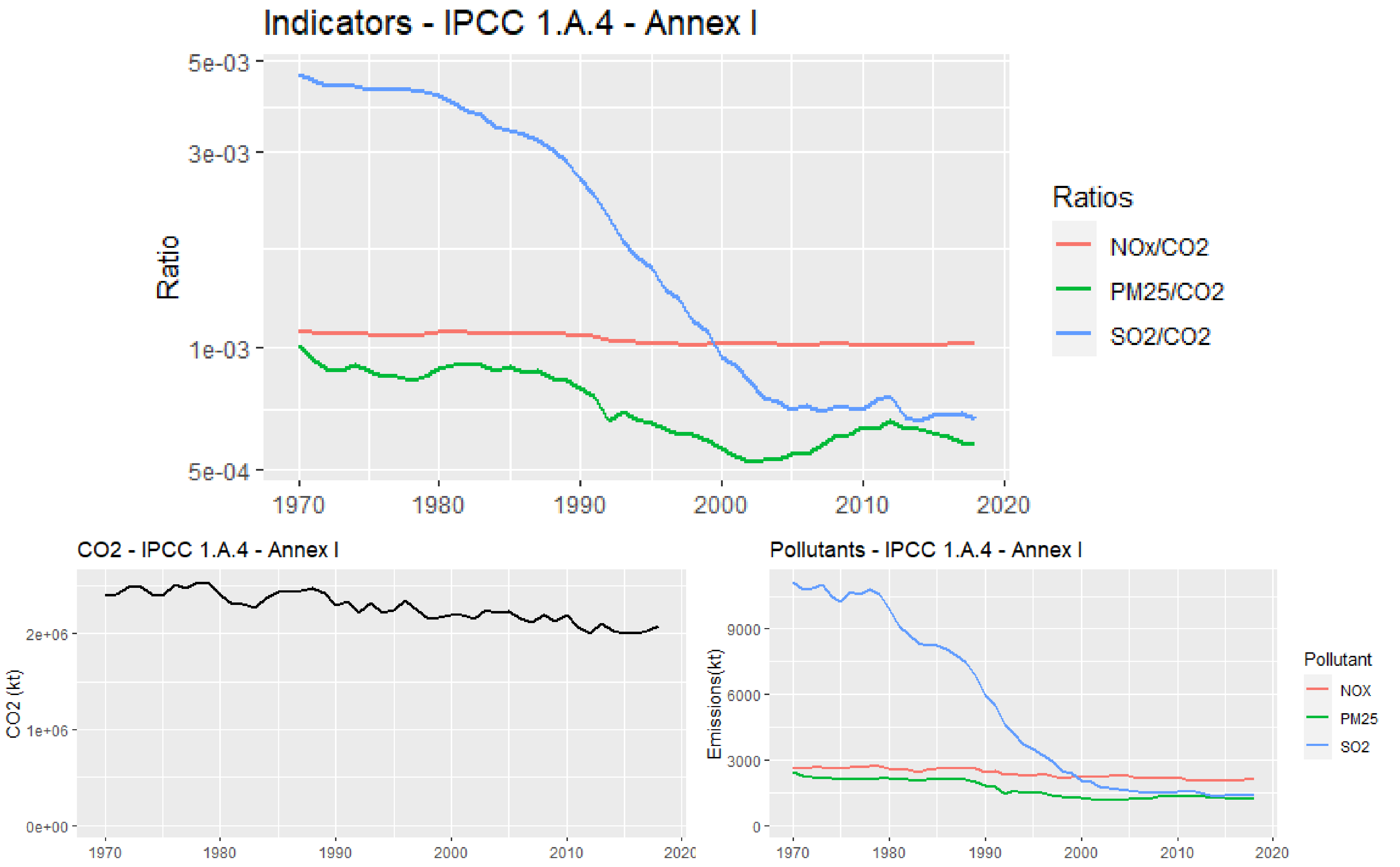

Figure S.22 – Top panel: Time series (1970-2018) of selected indicators in the IPCC 1.A.4 (Buildings) sector in FAI countries. Bottom left: CO_2 emissions (1970-2018) in 1.A.4 sector in FAI countries. Bottom right: emissions (1970-2018) of selected pollutants in 1.A.4 sector in FAI countries. Related to Figure 1.

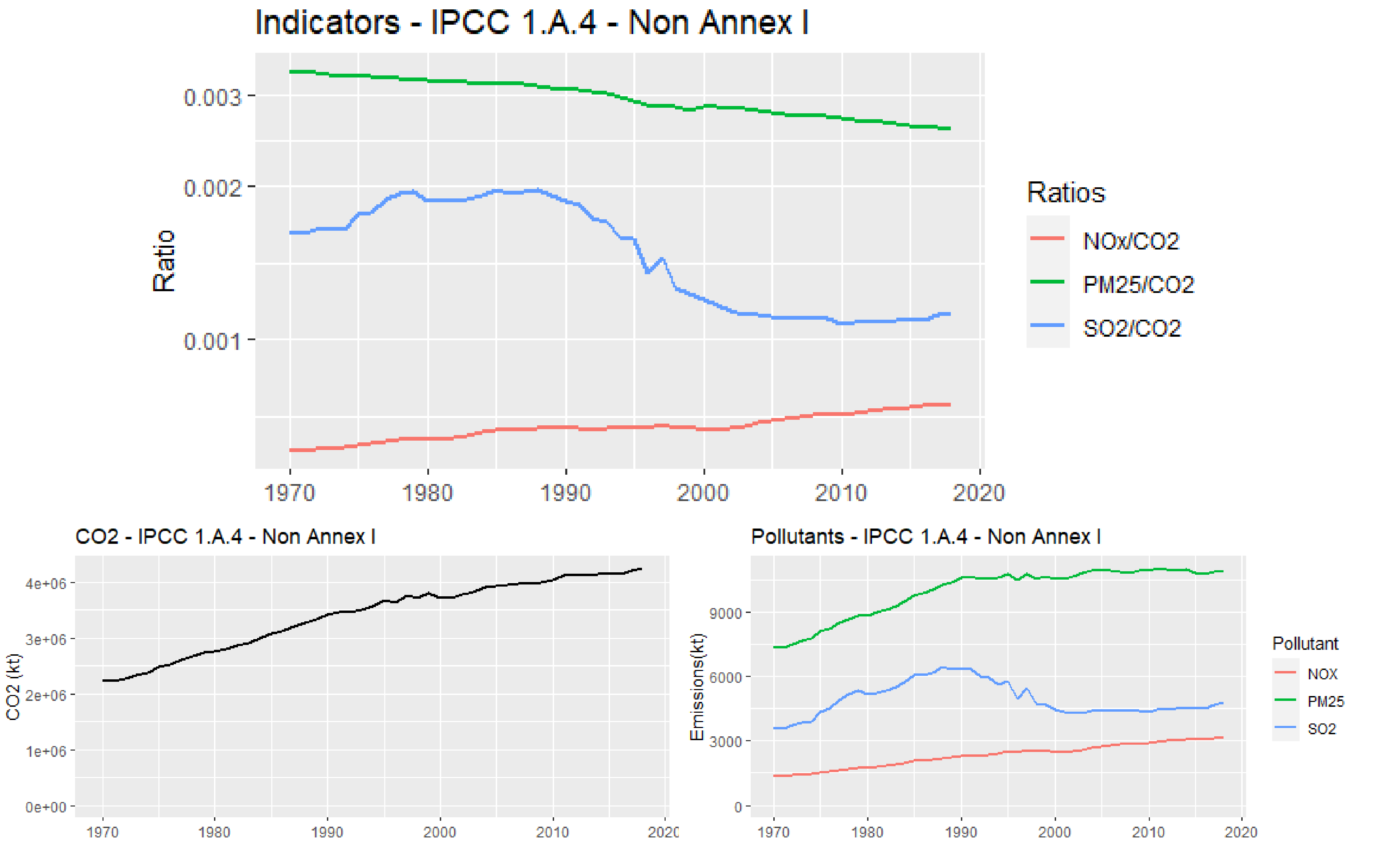

Figure S.23 – Top panel: Time series (1970-2018) of selected indicators in the IPCC 1.A.4 (Buildings) sector in FNAI countries. Bottom left: CO_2 emissions (1970-2018) in 1.A.4 sector in FNAI countries. Bottom right: emissions (1970-2018) of selected pollutants in 1.A.4 sector in FNAI countries. Related to Figure 1.

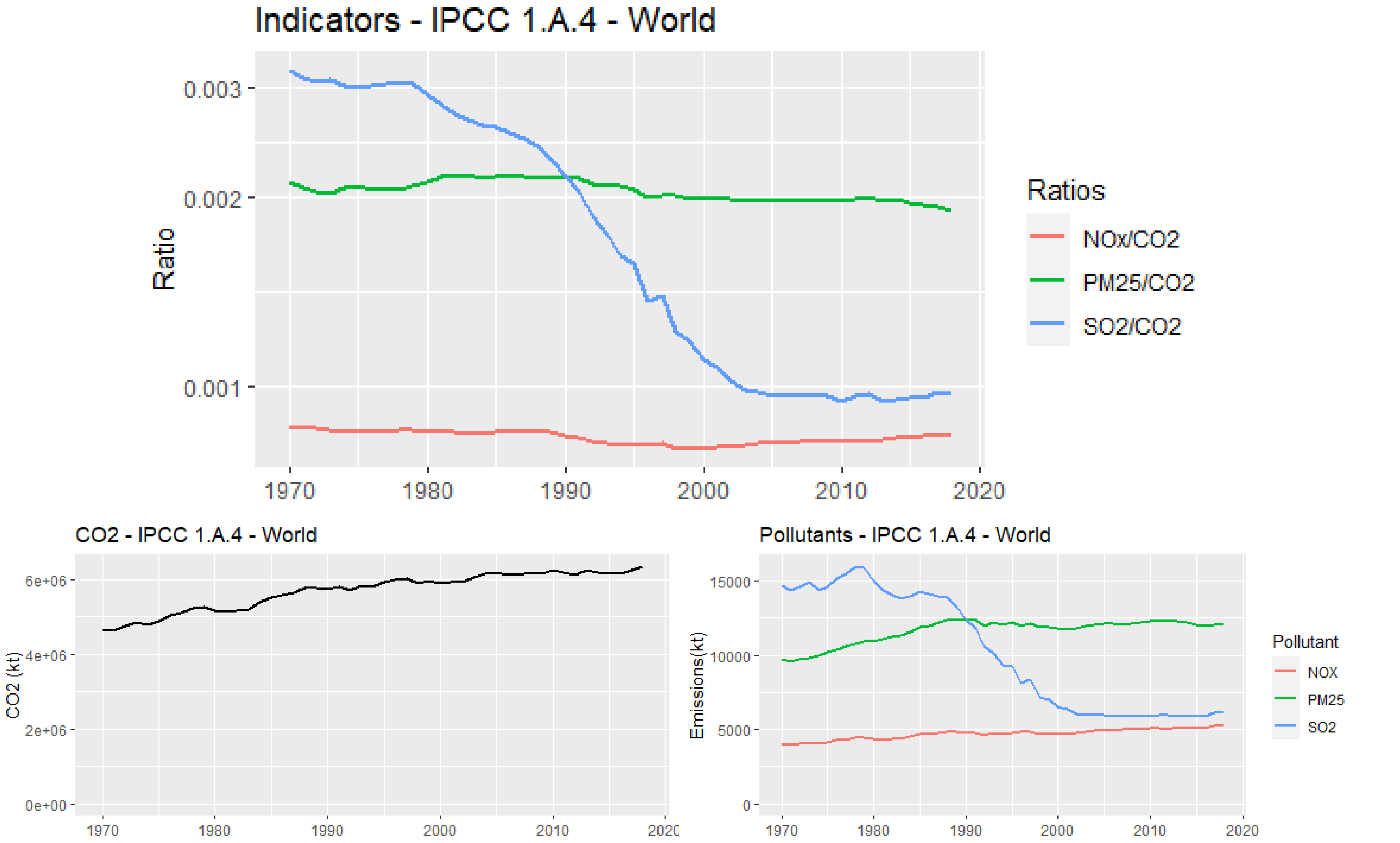

Figure S.24 – Top panel: Time series (1970-2018) of selected indicators in the IPCC 1.A.4 (Buildings) sector in all world countries. Bottom left: CO_2 emissions (1970-2018) in 1.A.4 sector in all world countries. Bottom right: emissions (1970-2018) of selected pollutants in 1.A.4 sector in all world countries. Related to Figure 1.
